# High-Fat Diet and Feeding Regime Impairs Number, Phenotype, and Cytotoxicity of Natural Killer Cells in C57BL/6 Mice

**DOI:** 10.3389/fnut.2020.585693

**Published:** 2020-11-27

**Authors:** Julia Spielmann, Wiebke Naujoks, Matthias Emde, Martin Allweyer, Heike Kielstein, Dagmar Quandt, Ina Bähr

**Affiliations:** ^1^Institute of Anatomy and Cell Biology, Medical Faculty of Martin Luther University Halle-Wittenberg, Halle (Saale), Germany; ^2^School of Medicine, College of Medicine, Nursing and Health Sciences, Regenerative Medicine Institute (REMEDI) at CÚRAM Centre for Research in Medical Devices, National University of Ireland Galway, Galway, Ireland

**Keywords:** NK cells, overweight, obesity, cytotoxicity, NK cell receptors, NK cell subsets, cancer, mice

## Abstract

Overweight and obesity are major public health challenges worldwide. Obesity is associated with a higher risk for the development of several cancer types, but specific mechanisms underlying the link of obesity and cancer are still unclear. Natural killer (NK) cells are circulating lymphoid cells promoting the elimination of virus-infected and tumor cells. Previous investigations demonstrated conflicting results concerning the influence of obesity on functional NK cell parameters in small animal models. The aim of the present study was to clarify potential obesity-associated alterations of murine NK cells *in vivo*, implementing different feeding regimes. Therefore, C57BL/6 mice were fed a normal-fat diet (NFD) or high-fat diet (HFD) under restrictive and *ad libitum* feeding regimes. Results showed diet and feeding-regime dependent differences in body weight, visceral fat mass and plasma cytokine concentrations. Flow cytometry analyses demonstrated significant changes in total cell counts as well as frequencies of immune cell populations in peripheral blood comparing mice fed NFD or HFD in an *ad libitum* or restrictive manner. Mice fed the HFD showed significantly decreased frequencies of total NK cells and the mature CD11b^+^CD27^+^ NK cell subset compared to mice fed the NFD. Feeding HFD resulted in significant changes in the expression of the maturation markers KLRG1 and CD127 in NK cells. Furthermore, real-time PCR analyses of NK-cell related functional parameters in adipose tissue revealed significant diet and feeding-regime dependent differences. Most notable, real-time cytotoxicity assays demonstrated an impaired cytolytic activity of splenic NK cells toward murine colon cancer cells in HFD-fed mice compared to NFD-fed mice. In conclusion, our data demonstrate that feeding a high-fat diet influences the frequency, phenotype and function of NK cells in C57BL/6 mice. Interestingly, restricted feeding of HFD compared to *ad libitum* feeding resulted in a partial prevention of the obesity-associated alterations on immune cells and especially on NK cells, nicely fitting with the current concept of an advantage for interval fasting for improved health.

## Introduction

Obesity is a complex public health problem affecting all age and socio-economic groups worldwide. In 2016, 39% of adults (1.9 billion) were overweight and 13% (650 million) of adults were obese (1). Overweight and obesity are main risk factors for the development of several diseases, like diabetes, coronary heart disease, as well as kidney and musculoskeletal disorders (2–5). Moreover, obese individuals have an increased susceptibility to infections and a higher risk for numerous cancer types, like postmenopausal breast, colorectal, kidney, esophageal and pancreatic cancer (6–8). In addition, obesity is associated with an increased mortality risk resulting in more people dying from obesity than from underweight (1, 9). Although the role of obesity in cancer development is not completely understood, several biological mechanisms for the association between excess body weight and cancer, like hyperinsulinemia, increased levels of sex hormones, alterations in microenvironment or dietary factors have been discussed (10). In addition, the abnormal increased number of adipocytes and immune cells in adipose tissue in obesity leads to an altered secretion pattern of adipokines as well as a high release of pro-inflammatory cytokines, leading to a subclinical systemic low-grade inflammation (11). Previous investigations demonstrated an impaired functionality of several immune cells, like T and B lymphocytes as well as monocytes under obese conditions (12–14). Moreover, animal and human studies described an impaired NK cell physiology in obesity (15–21).

NK cells are members of the innate lymphoid cell family that rapidly recognize and kill virally infected and malignant transformed cells without prior sensitization via different mechanisms. Human NK cells are characterized by the surface expression of CD56 and the absence of the T cell marker CD3. According to the surface expression of CD56 and CD16, human NK cells are commonly divided in two major subpopulations, the more cytotoxic CD56^dim^CD16^bright^ NK cells and the more immune regulatory CD56^bright^CD16^dim^ NK cells (22).

In contrast to humans, NK cells in mice are commonly identified by the presence of NKp46 (CD335), CD49b (DX5), and NK1.1 (CD161) gated on CD3 negative cells. During maturation, murine NK cells upregulate the expression of CD11b and downregulate CD27 expression. Based on the surface density of CD11b and CD27, four murine NK cell subsets, namely CD11b^low^CD27^low^, CD11b^low^CD27^high^, CD11b^high^CD27^high^, and CD11b^high^CD27^low^, are currently discriminated according to the maturation stages (23, 24). The CD11b^high^CD27^high^ NK cell subsets was described to exhibit the highest capacity for cytotoxicity and cytokine secretion (23, 25).

NK cells mediate cellular cytotoxicity induced by a release of cytotoxic granules, containing perforin and granzymes, or induce apoptosis of target cells via interaction with the death receptors Fas ligand and TRAIL (tumor necrosis factor related apoptosis inducing ligand) (26–28). In addition, activated NK cells secrete numerous cytokines, like interferon (IFN)-γ or tumor necrosis factor (TNF)-α, in order to co-stimulate other cells of the immune system (22). The NK cell-mediated target cell killing and cytokine secretion is controlled by a balanced expression of inhibitory, costimulatory, and activating receptors (29–31). In humans, most relevant activating NK cell receptors comprise the natural cytotoxicity receptors (NCRs) NKp30, NKp44, and NKp46, the short-tail members of killer immunoglobulin-like receptors (KIRs) and the natural killer group (NKG) 2D receptor (32). Inhibitory NK cell receptors are the killer cell lectin-like receptor (KLR) subfamily G1, the long-tail members of the KIR family, as well as the NKG2A receptor (32, 33).

Although murine NK cells show similarities to human NK cells regarding the NK cell receptor expression profile, like the NKG2D and NKp46 expression, there are also important differences in NK cell receptor repertoire comparing both species. In particular, mice lack the expression of NKp44 and NKp30, although NKp30 was demonstrated to be expressed as an unexpressed pseudogene (30, 34). Moreover, the functional analog for human KIRs is the lectin-like Ly49 receptor family in mice (33, 35).

Previous *in-vitro* and *in vivo*-investigations demonstrated that several adipokines, like leptin, adiponectin, and interleukin(IL)-6, influences the functional activity of rodent and human NK cells (18, 36–38). In addition, numerous animal and human studies demonstrated that obesity is associated with alterations in number, phenotype, cytotoxicity and cytokine secretion of NK cells in rats and mice (17–21, 38–40).

Until now, most studies analyzing the effect of obesity on NK cell biology in mice were performed on C57BL/6 mice with diet-induced obesity under an *ad libitum* feeding regime (38). Previous studies demonstrated that the use of different feeding regimes, like time- or caloric restricted feeding, influences the development of obesity in mice and, consequently, metabolic and immunological parameters (41–44). Until now, no data exist on the impact of different feeding regimes on NK cell physiology in diet-induced obese mice. In addition, to our knowledge, there is no study that investigated the NK cell surface marker expression on murine NK cells subsets of obese mice. Therefore, aim of the present study was the characterization of the number, subset distribution, expression of NK cell surface receptors on total NK cells and NK cell subsets in peripheral blood as well as cytotoxicity of splenic NK cells in C57BL/6 mice fed a control or high-fat diet *ad libitum* or in a restrictive feeding regime.

## Materials and Methods

### Mouse Husbandry, Feeding Regimes, and Experimental Setup

Six weeks old male C57BL/6 mice (*n* = 34) were maintained on a 12 h light/12 h dark cycle with free access to pelleted food and water under controlled conditions at 23 ± 2°C and 55 ± 5% relative humidity. After 1 week of acclimatization under *ad libitum* feeding with regular rodent chow (Altromin, Lage, Germany), mice were each randomized into four groups and subsequently housed individually. Mice received either a normal-fat diet (NFD; 10% fat; D12450J, Research Diets, New Brunswick, USA; *n* = 14), or to induce obesity, a high-fat diet (HFD; 60% fat; D12492 – matches the sucrose calories in D12450J, Research Diets; *n* = 21) for a duration of 17 weeks. Additionally, mice were fed the particular diet either *ad libitum* (*n* = 7 for NFD; *n* = 10 for HFD) or restrictively (*n* = 7 for NFD; *n* = 11 for HFD). As C57BL/6 mice have previously been shown to be partly obesity-resistant, a higher number of mice was used in both HFD-fed groups (45, 46). Mice fed the restrictive feeding regime received 90% of the daily food intake of the corresponding *ad libitum* group. Food intake of all experimental groups was documented daily and the respective food amount for the restrictive fed groups was calculated daily. The diets were provided every day at the same time – at the beginning of the active phase of mice. Daily intake of energy, fat, protein, and carbohydrate was calculated using the daily food intake and data of diet composition given by the manufacturer. Body weight was determined every week. All research and animal care procedures were approved by the local animal care committee (reference number 42502–2-1341 MLU).

### Mouse Anesthesia, Sacrificing, and Sample Collection

Seventeen weeks after starting the feeding with NFD or HFD, final body weight was determined. Animals were sacrificed under general isoflurane inhalation anesthesia (1.5–2.0% v/v in O_2_) by puncture of the cardiac ventricle and exsanguination. Blood was withdrawn, mixed with 10 μl ethylenediaminetetraacetic acid tetrasodium salt (EDTA) anticoagulant and stored on crushed ice. A fraction of blood (~500 μl) was used for following flow cytometric analysis. Plasma was obtained by centrifugation and stored at −80°C for cytokine analysis. Visceral adipose tissue and spleen were removed and weights were determined. Spleens of mice were kept in splenocyte culture medium consisting of RPMI medium (Corning) supplemented with 10% FBS, 1% penicillin-streptomycin and 50 μM 2-mercaptoethanol (Merck) at 37°C and were immediately used for cytotoxicity assays. Samples of visceral adipose tissue were frozen in liquid nitrogen and stored at −80°C for further analyses.

### Multiplex Immunoassay of Murine Plasma Cytokines

Measurements of plasma cytokine concentrations were performed using a multiplex immunoassay kit (High Sensitivity 5-Plex Mouse ProcartaPlex™ Panel; Thermo Fisher Scientific, Darmstadt, Germany) following the manufacturer's instructions. Cytokine levels were determined using the LiquiChip luminex 200 system (Qiagen, Hilden, Germany) and the Procartaplex-analyst 1.0 software (Affymetrix, eBioscience, San Diego, USA).

### Multicolor Flow Cytometry of Peripheral Blood Immune Cells

Murine whole blood was stained with an appropriate combination of fluorochrome-conjugated monoclonal antibodies to analyze different leukocyte subsets and NK cell receptor surface expression by flow cytometry. Antibodies for surface staining are presented in Supplementary Table 1. Initially, 70 μl of whole blood was mixed with 50 μl antibody master mix and incubated for 15 min protected from light. Next, samples were incubated with red blood cell (RBC) lysis buffer (c.c. pro GmbH, Oberdorla, Germany) for 10 min to remove RBCs and to obtain leukocytes. Samples were washed twice with FACS (fluorescence activated cell sorting) buffer consisting of phosphate buffered saline (PBS; Merck); 1% FBS, 2 mM EDTA, and 0.05% sodium azide (both Carl Roth, Karlsruhe, Germany). Finally, all samples were resuspended in FACS buffer and kept at 4°C in the dark until flow cytometric analysis.

In order to clearly distinguish the different immune cell populations in each tube and to identify only specific surface molecules on NK cells, a backbone antibody staining against leukocytes (CD45), T lymphocytes (CD3) and NK cells (CD335) was used in each panel. One tube only contained these three antibodies and served as a Fluorescence Minus One (FMO) control. For determining absolute counts of leukocytes in murine blood, Trucount tubes (Trucount™ Tubes, BD Biosciences, Sa Jose, USA) with a known number of fluorescent beads were used instead of normal FACS tubes.

All samples were measured by using the flow cytometry analyzer BD LSRFortessa™ (Becton Dickinson, Heidelberg, Germany). Measured data were analyzed using BD FACS Diva™ software version 7.0 (Becton Dickinson) and FlowJo version 8.7 (FlowJo LLC, Ashland, USA). For the immunophenotyping of NK cells by flow cytometric analysis 50,000–100,000 events were measured per panel. Identification of immune cell populations, NK cell subsets and NK cell phenotyping of surface markers was based on the gating strategies depicted in Supplementary Figure 1. Representative flow cytometric dot plots or histograms for multiple different surface markers are shown in Supplementary Figure 2. Percentages are given where gating for positive cells was feasible otherwise median fluorescence intensity for individual markers are plotted.

### Tumor Cell Culture, NK Cell Purification, and Real-Time Cytotoxicity Assay

The CT26.WT murine colon carcinoma target cell line was cultured with RPMI 1640 (Corning, Corning, USA) supplemented with 10% of fetal bovine serum (FBS; Merck, Darmstadt, Germany), 1% penicillin/streptomycin (10,000 U/ml penicillin; 10 μg/ml streptomycin; Sigma-Aldrich Inc., St. Louis, USA) and 1% sodium-pyruvate (100 mM). Cells were maintained under standard cell culture conditions at 37°C, 5% CO_2_ and a humidified atmosphere.

Prior to the isolation of splenic effector NK cells, a single cell suspension of murine splenocytes from each mouse spleen was prepared. Therefore, spleens of mice were cut into small pieces. After adding of MACS puffer (MACS rinsing solution and MACS BSA stock solution, 20:1, both Miltenyi Biotec), the tissue pieces were transferred into a 40 μm cell strainer placed in a centrifuge tube and gently minced with a syringe plunger. After washing and centrifugation, the final pellet was resuspended in MACS buffer and cell number was determined. Primary splenic NK cells were isolated by negative magnetic activated cell sorting (MACS) using a murine NK cell isolation kit (Miltenyi Biotec) according to manufacturer's instructions.

Cytotoxicity assays were performed using the real-time cell analysis (RTCA) systems instruments (xCELLigence, ACEA, Biosciences, San Diego, USA).

The murine target cells CT26.WT were seeded on the E-plates with a cell number of 15,000 cells/well in 200 μl splenocyte medium. After culturing CT26.WT target cells for 24 h, the freshly isolated NK cells were added at an effector:target (E:T) ratio of 70:1 in a volume of 100 μl. In addition, 500 U/ml recombinant murine IL-2 (Reprokine, Rehovot, Israel) was added. One well of each E-plate was constantly loaded with medium only and served as a medium control. After adding of NK cells, data were collected every minute for 2 h, followed by measurements with an interval of 15 min until the experiment was finally stopped after 45 h. Each sample was usually analyzed in double or triple determination. The cytolytic activity of the effector NK cells at a given time point was determined by using the calculated normalized cell index according to the instructions by the manufacturer.

### Real-Time RT-PCR Analyses

Total RNA of frozen adipose tissue was extracted using the RNeasy® Lipid Tissue Mini Kit (Qiagen, Hilden, Germany) according to the manufacturer's instructions. Subsequently, total RNA was reverse transcribed into cDNA using the ThermoScript RT-PCR system (Thermo Fisher Scientific Inc., Darmstadt, Germany) according to the supplier's recommendations. Quantitative real-time PCR was performed using SYBR Green Fluorescein Mix (BioRad, München, Germany) and the qTOWER3 thermocycler (Analytik Jena AG, Jena, Deutschland). Relative expression levels of samples were determined using the ΔΔCt method, using peptidylprolyl isomerase A (PPIA) as housekeeping gene. Details of primers pairs are listed in Supplementary Table 2. Amplification efficiency for each primer pair was calculated from the slope of the standard curve using different primer dilutions.

### Statistical Analyses

All statistical analyses were performed using the GraphPad Prism software version 6.07 (La Jolla, USA). Outliers were identified using GraphPad Prism's robust regression and outlier removal (ROUT) method followed by testing for normal distribution and homogeneity of variances. To assess the main effect of the two investigated independent factors “diet” and “feeding regime” as well as a possible interaction between them, we performed two-way ANOVA analyses. Significant results of the two-way-ANOVA effects of the factor “diet” were indicated with asterisks (*P* values: ^*^P ≤ 0.05, ^**^P ≤ 0.01, and ^***^P ≤ 0.001). Significances of two-way ANOVA analyses regarding the main factor “feeding regime” are indicated with precise *P* values within the text sections of the results. To compare means of the four individual mouse groups, we subsequently performed a *post-hoc* Tukey's multiple comparison test. Significant results of the *post-hoc* test are indicated using different letters (a, b, c, d). *P* ≤ 0.05 was considered to denote significant differences. Results are presented as means ± standard error of the mean (SEM).

## Results

### Dietary Intake

The type of the diet as well as the feeding regime had significant influence on food, energy and fat intakes as well as on carbohydrate and protein intakes (Supplementary Table 3).

In both the NFD and HFD groups, intake of all nutritional parameters was significantly lower in mice fed the restrictive feeding regime compared to mice fed *ad libitum*, except for the daily fat intake of NFD-fed mice, which was not significantly influenced by the feeding regime. Although the daily food intake of mice fed the HFD *ad libitum* or restrictive was significantly lower compared to the corresponding NFD-fed groups, the energy intake was higher in HFD-fed mice compared to NFD-fed mice in both feeding regimes due to the high energy content of the HFD (Supplementary Table 3). Moreover, mice fed a HFD *ad libitum* or restrictive had a significantly increased daily intake of fat and protein and a significantly decreased daily intake of carbohydrates compared to the corresponding NFD control groups (Supplementary Table 3).

### Body Weight Gain and Visceral Fat Mass

**Figure 1:**
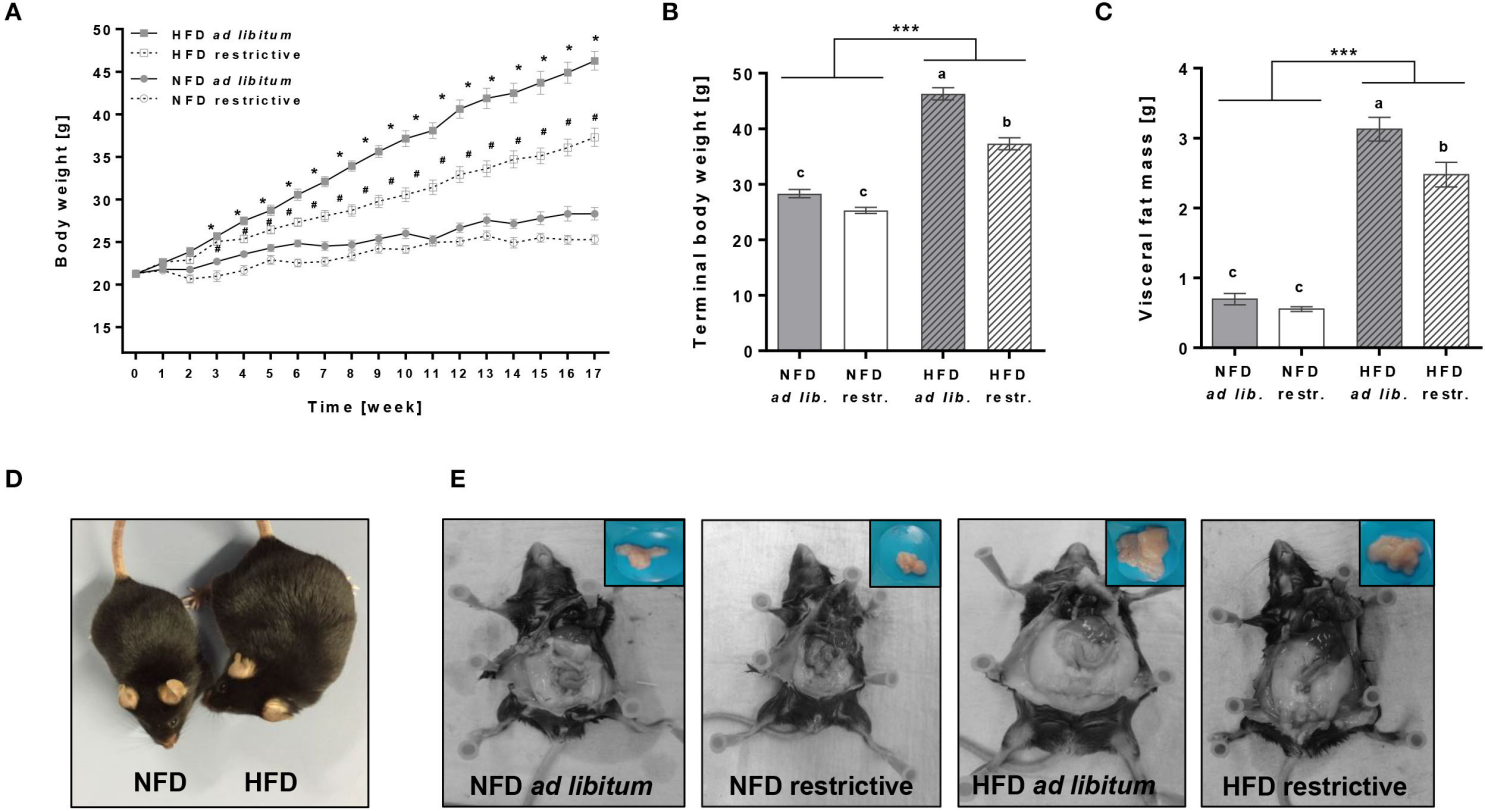
Effects of high-fat diet (HFD) and normal-fat diet (NFD) under *ad libitum* (*ad lib*.) and restrictive (restr.) feeding regimes on male C57BL/6 mice. Body weight gain **(A)**, terminal body weight **(B)** and visceral fat mass **(C)** after 17 weeks of feeding the diets. Representative pictures for visual differences in body weight of C57BL/6 mice fed NFD *ad libitum* and HFD *ad libitum* **(D)** and visceral fat mass of C57BL/6 mice fed NFD *ad libitum* or restrictive and HFD *ad libitum* or restrictive **(E)** after 17 weeks of feeding the diets. *P < 0.05, HFD *ad libitum* compared to NFD *ad libitum* group. #*P* < 0.05, HFD restrictive compared to NFD restrictive group. Means with different letters are significantly different according to *post-hoc* Tukey's multiple comparison test results (*P* ≤ 0.05). ***P ≤ 0.001, two-way ANOVA, NFD-fed groups compared to HFD-fed groups. Data are presented as mean ± SEM.

*ad libitum* as well as restrictive HFD feeding significantly increased the body weight of mice from week 3 onward compared to the respective NFD groups (Figures 1A,B, Supplementary Table 4). Moreover, terminal body weights and visceral fat mass were significantly increased in mice fed the HFD *ad libitum* or restrictive compared to the corresponding NFD-fed group (Figures 1B,C, Supplementary Table 4). Restrictive feeding resulted in significant lower terminal body weights and visceral fat mass of HFD-fed mice, but not in NFD-fed mice (Figures 1B,C, Supplementary Table 4). Figures 1D,E demonstrate representative pictures showing visual differences in terminal body weights and visceral fat mass of NFD or HFD-fed C57BL/6 mice.

### Plasma Cytokine Concentrations

**Figure 2:**
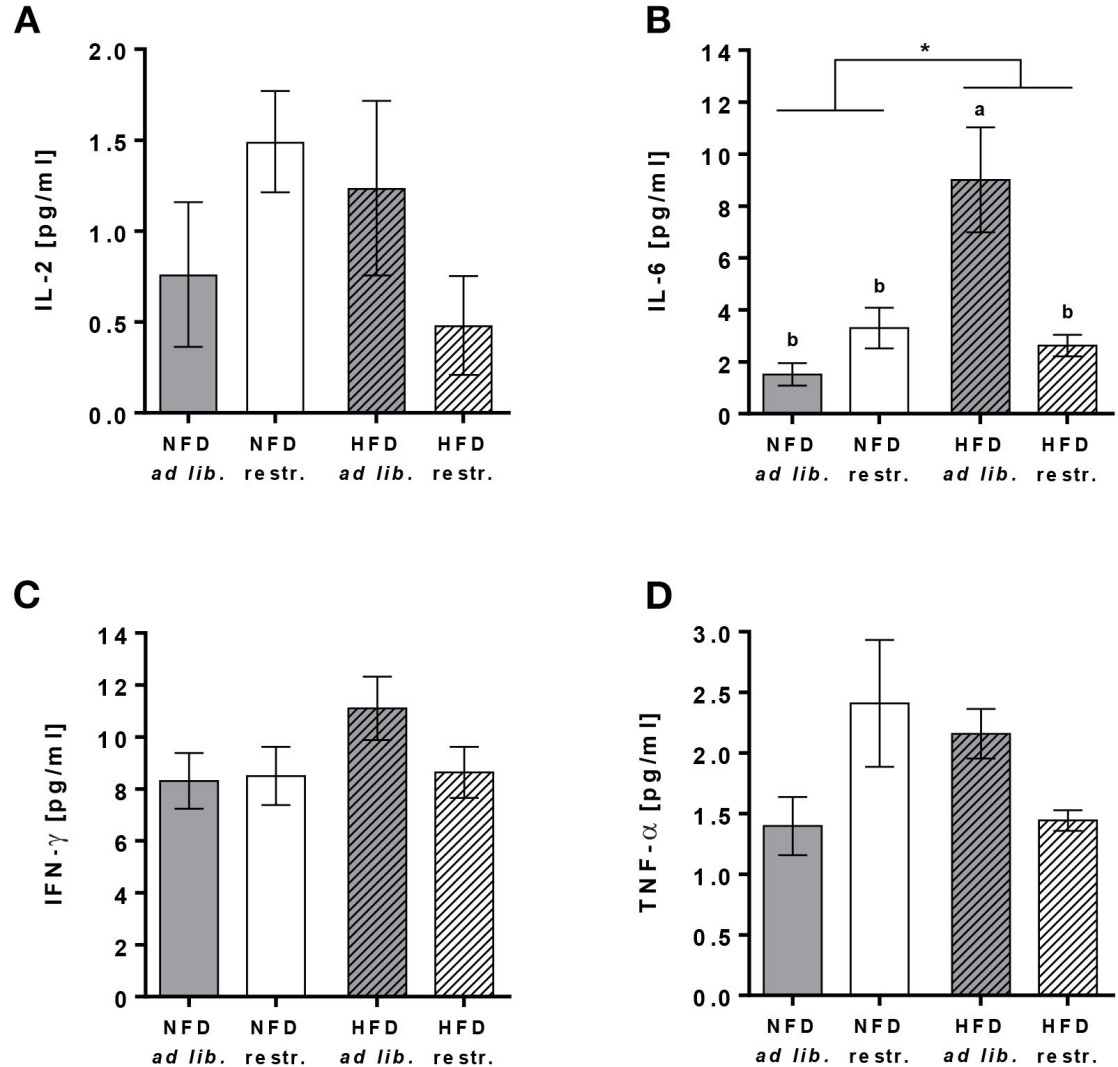
Multiplex analyses of plasma cytokine concentrations of interleukin (IL)-2 **(A)**, IL-6 **(B)**, interferon (IFN)-γ **(C)** and tumor necrosis factor (TNF)-α **(D)** in C57BL/6 mice fed a high-fat diet (HFD) and normal-fat diet (NFD) under *ad libitum* (*ad lib*.) and restrictive (restr.) feeding regimes. Means with different letters are significantly different according to *post-hoc* Tukey's multiple comparison test results (*P* ≤ 0.05). *P ≤ 0.05, two-way ANOVA, NFD-fed groups compared to HFD-fed groups. Data are presented as mean ± SEM.

Plasma concentrations of IL-6 were significantly increased in mice fed the HFD *ad libitum* compared to mice fed the NFD *ad libitum*, whereas mice received diets restrictively showed no differences in IL-6 plasma concentrations (Figure 2B, Supplementary Table 4). In addition, plasma IL-6 concentrations were significantly lower in mice fed the HFD *ad libitum* compared to the corresponding group fed the HFD in a restrictive manner (Figure 2B, Supplementary Table 4). No changes in plasma concentrations of IL-2, IFN-γ and TNF-α were detected comparing all experimental groups (Figures 2A,C,D, Supplementary Table 4).

### Analyses of Immune Cell Populations Other Than NK Cells in Peripheral Blood

**Figure 3:**
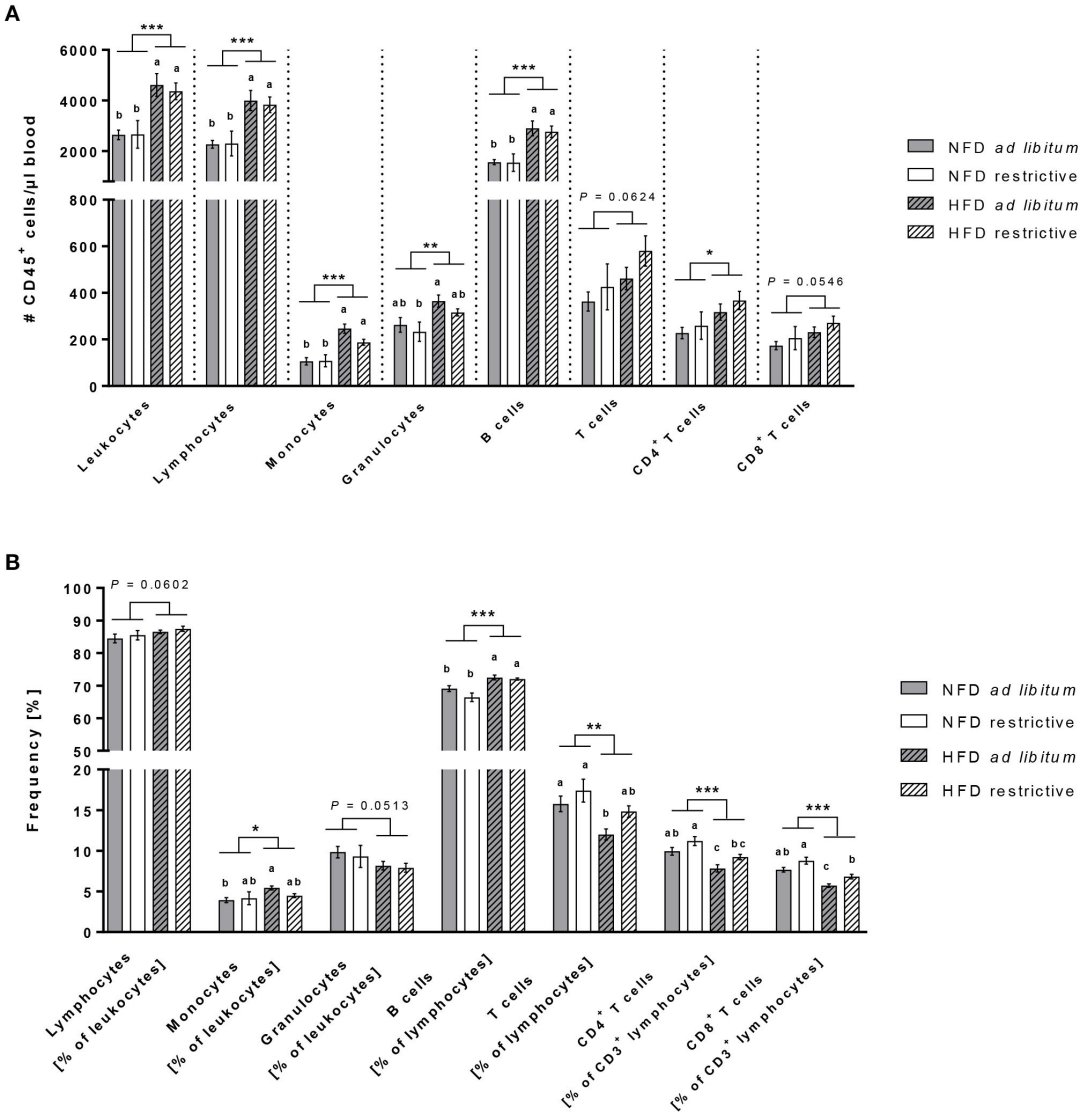
Flow cytometric analyses of immune cell subsets in blood. Absolute cell counts **(A)** and frequencies **(B)** of immune cell populations in whole blood samples of C57BL/6 mice fed either a high-fat diet (HFD) or normal-fat diet (NFD) under *ad libitum* (ad lib.) and restrictive (restr.) feeding regimes. Means with different letters are significantly different according to *post-hoc* Tukey's multiple comparison test results (*P* ≤ 0.05). *P ≤ 0.05 and exact *P*-values within 0.05 ≤ *P* ≤ 0.1, two-way ANOVA, NFD-fed groups compared to HFD-fed groups. Data are presented as mean ± SEM. ^**^P ≤ 0.01; ^***^P ≤ 0.001.

The hierarchical pathways of flow cytometric analyses of immune cell populations are depicted in Supplementary Figure 3. Analyses of total blood cell counts revealed an almost doubling of leukocytes in HDF-fed mice compared to NFD-fed mice regardless of the feeding regime (Figure 3A). Dissecting the different immune cell populations, B cells and monocytes followed by granulocytes were the far most significantly increased cell populations upon HDF feeding (Figure 3A). In addition, T lymphocytes were also affected by dietary feeding, CD3^+^CD4^+^ helper T cells were significantly increased and CD3^+^CD8^+^ cytotoxic T cells were slightly increased in HFD-fed compared to NFD-fed mice (Figure 3A).

Analyses of the proportional distribution of the respective immune cell populations by means of percentages elucidated some interesting and divergent findings from the total cell counts (Figure 3B). The proportions of B cells and monocytes (in % of CD45^+^ leukocytes) were robustly higher in HFD-fed mice compared to NFD-fed mice with a significant difference between both *ad libitum*-fed groups (Figure 3B). In contrast to the elevated cell counts of granulocytes in HFD-fed mice, the percentages of granulocytes (in % of CD45^+^ leukocytes) in the blood was slightly decreased in HFD-fed mice (Figure 3B). The proportions of total T cells (in % of CD45^+^ lymphocytes) as well as both CD4^+^ and CD8^+^ T cell subsets (in % of CD3^−^ lymphocytes) analyzed from a CD45^+^ lymphocyte pregating showed decreased frequencies of > 20% difference in HFD-fed animals compared to NFD-fed animals (Figure 3B). In addition and exclusively so for T cells, restrictive feeding led to increased frequencies of CD4^+^ and CD8^+^ T cells (in % of CD3^−^ lymphocytes) compared to *ad libitum*-fed mice independent of the diet (Figure 3B).

### Investigations on NK Cell Subsets in Peripheral Blood

In addition to the mentioned immune cell populations above, the cell counts and frequencies of total primary murine NK cells (in % of CD45^+^ lymphocytes) as well as the frequencies of the four murine NK cell subsets CD11b^−^CD27^−^, CD11b^−^CD27^+^, CD11b^+^CD27^+^, and CD11b^+^CD27^−^ (in % of total NK cells) were determined.

**Figure 4:**
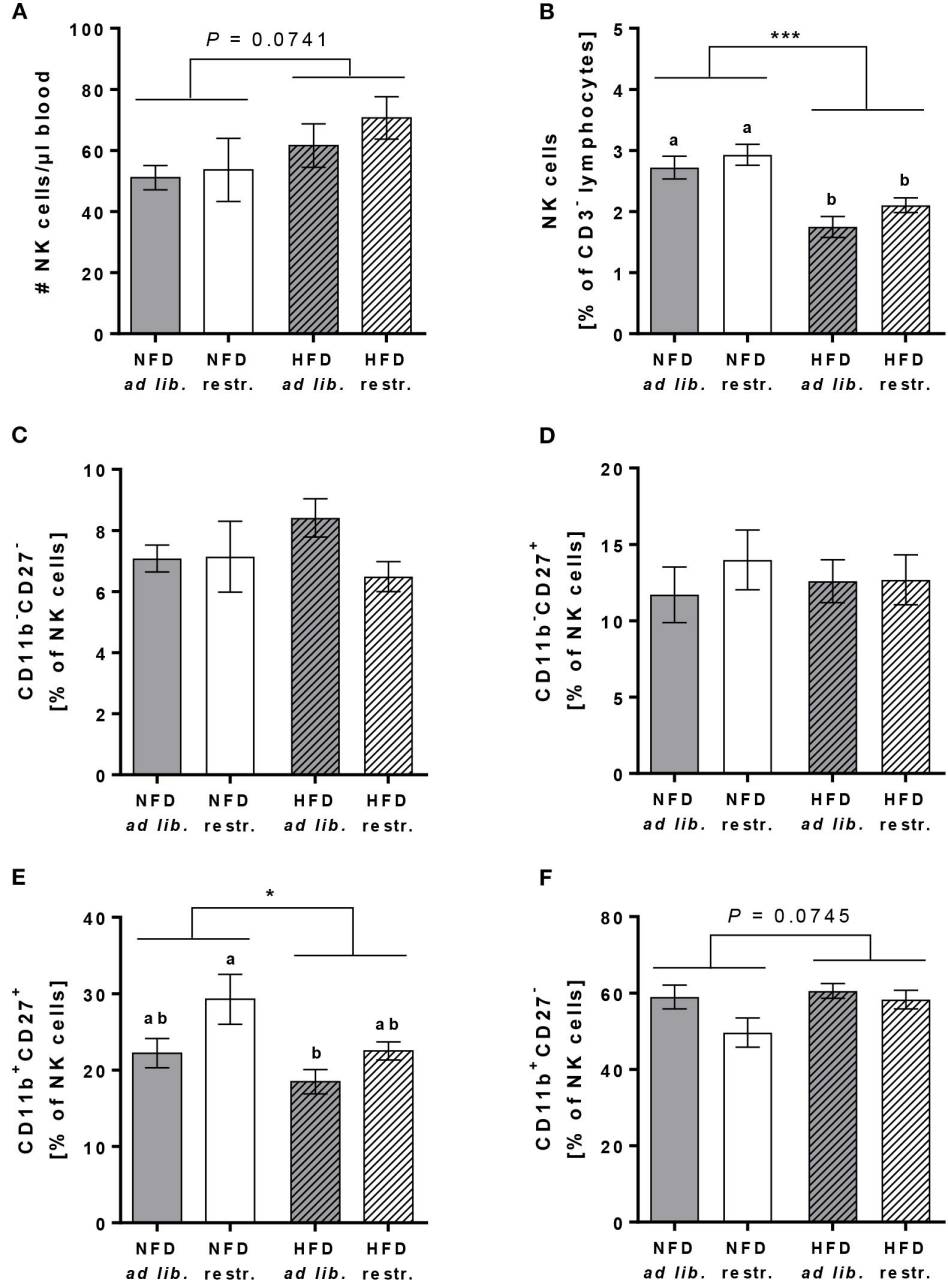
Flow cytometric analyses of NK cells and NK cell subsets in whole blood samples of C57BL/6 mice fed either a high-fat diet (HFD) or normal-fat diet (NFD) under *ad libitum* (*ad lib*.) and restrictive (restr.) feeding regimes. Concentration of total NK cells **(A)**, expressed as absolute cell number per μl, and percentages of total NK cells **(B)**. Percentages of NK cell subsets with different surface expression profiles of CD11b and CD27 **(C–F)**. ^*^P ≤ 0.05; ^***^P ≤ 0.001 and exact *P*-values within 0.05 ≤ *P* ≤ 0.1, two-way ANOVA, NFD-fed groups compared to HFD-fed groups. Means with different letters are significantly different according to *post-hoc* Tukey's multiple comparison test results (*P* ≤ 0.05). Data are presented as mean ± SEM.

The absolute NK cell numbers per μl blood was slightly increased in HFD-fed mice compared to NFD-fed mice, whereas the frequency of NK cells (in % of CD45^+^ lymphocytes) was significantly decreased in HFD-fed mice in both the *ad libitum*- and restrictive fed groups (Figures 4A,B). There were no significant differences in the frequencies of the CD11b^−^CD27^−^, CD11b^−^CD27^+^ and the CD11b^+^CD27^−^ NK cell subsets (in % of total NK cells) between the single diet groups (Figures 4C,D,F). In contrast, HFD-fed mice showed significantly reduced frequencies of the CD11b^+^CD27^+^ NK cell subset (in % of total NK cells) with a significant difference between HFD *ad libitum-* and NFD restrictive-fed mice (Figure 4E). Results demonstrated a significant increase of the CD11b^+^CD27^+^ NK cell subset (in % of total NK cells) in restrictive-fed mice compared to *ad libitum*-fed mice independent of the diet (*P* = 0.0071). In contrast, the frequency of the considered most mature stage, the CD11b^+^CD27^−^ NK cell subset (in % of total NK cells), was significantly lower in restrictive-fed mice compared to *ad libitum*-fed mice fed independent of the diet (*P* = 0.0449).

### Phenotype Analyses of Total NK Cells and NK Cell Subsets in Peripheral Blood

**Figure 5:**
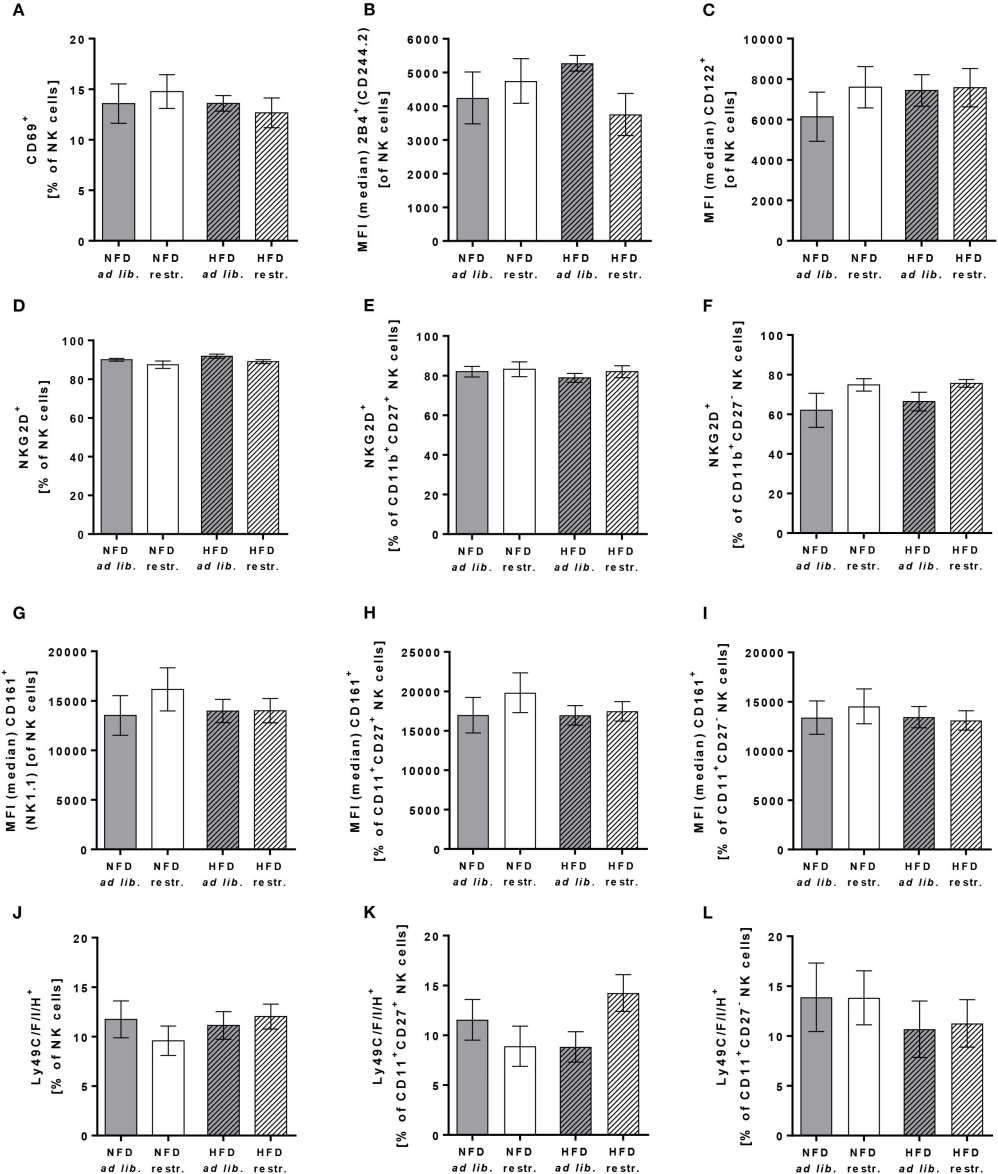
**(A–C)** Flow cytometric analyses of frequencies or median fluorescent intensities (MFIs) of total NK cells of C57BL/6 mice expressing the activation-associated receptor CD69 **(A)** or the co-activating receptors 2B4 **(B)** and CD122 **(C)**. **(D–L)** Frequencies or MFIs of total NK cells, CD11b^+^CD27^+^NK cells and CD11b^+^CD27^−^ NK cells of C57BL/6 mice expressing the activating receptor NKG2D **(D–F)**, the activating receptor CD161 **(G–I)** or the inhibitory and activating receptors Ly49C/F/I/H **(J–L)**. Mice were fed either a high-fat diet (HFD) or normal-fat diet (NFD) under *ad libitum* (*ad lib*.) or restrictive (restr.) feeding regimes. Data are presented as mean ± SEM.

To characterize the effect of HFD and different feeding regimes on NK cell phenotype, the surface expression of functional NK cell receptors with activating and inhibitory function as well as adhesion molecules and maturation markers were determined in peripheral blood of C57BL/6 mice. Results of flow cytometric analyses demonstrated that the expression of the activation-associated receptor CD69 and the co-activating receptors 2B4 and CD122 on total NK cells was similar in all experimental groups (Figures 5A–C). Analyses of the frequency of NKG2D expressing total NK cells demonstrated a significant reduction in restrictive-fed mice compared to the *ad libitum*-fed mice independent of the diet (*P* = 0.0346, Figure 5D). No significant differences were detected in NKG2D expression on CD11b^+^CD27^+^ NK cells and CD11b^+^CD27^−^ NK cells between the four experimental groups (Figures 5E,F). However, results demonstrated a significant increase of NKG2D expression in CD11b^+^CD27^−^ NK cells in restrictive-fed mice compared to *ad libitum*-fed mice independent of the diet (*P* = 0.0453).

For the C-type lectin-like receptor CD161 (also called NKRP1), five receptors have been described in mice: NKRP1-A, -B, -C, -F, -G with either activating (-A, -C, -F) or inhibitory function (B, G) (47). The antibody used in this study detects the NKRP1C receptor, which is an activating form of the CD161 receptor. The antibody used for staining of Ly49 receptors recognizes a common epitope of the inhibitory family members Ly49C, F and I as well as the activating family member Ly49H, but does not react with other Ly49 members. Flow cytometric analyses of CD161 and Ly49C/F/I/H surface expression revealed no significant differences in total NK cells, CD11b^+^CD27^+^ NK cells and CD11b^+^CD27^−^ NK cells (Figures 5G–L).

**Figure 6:**
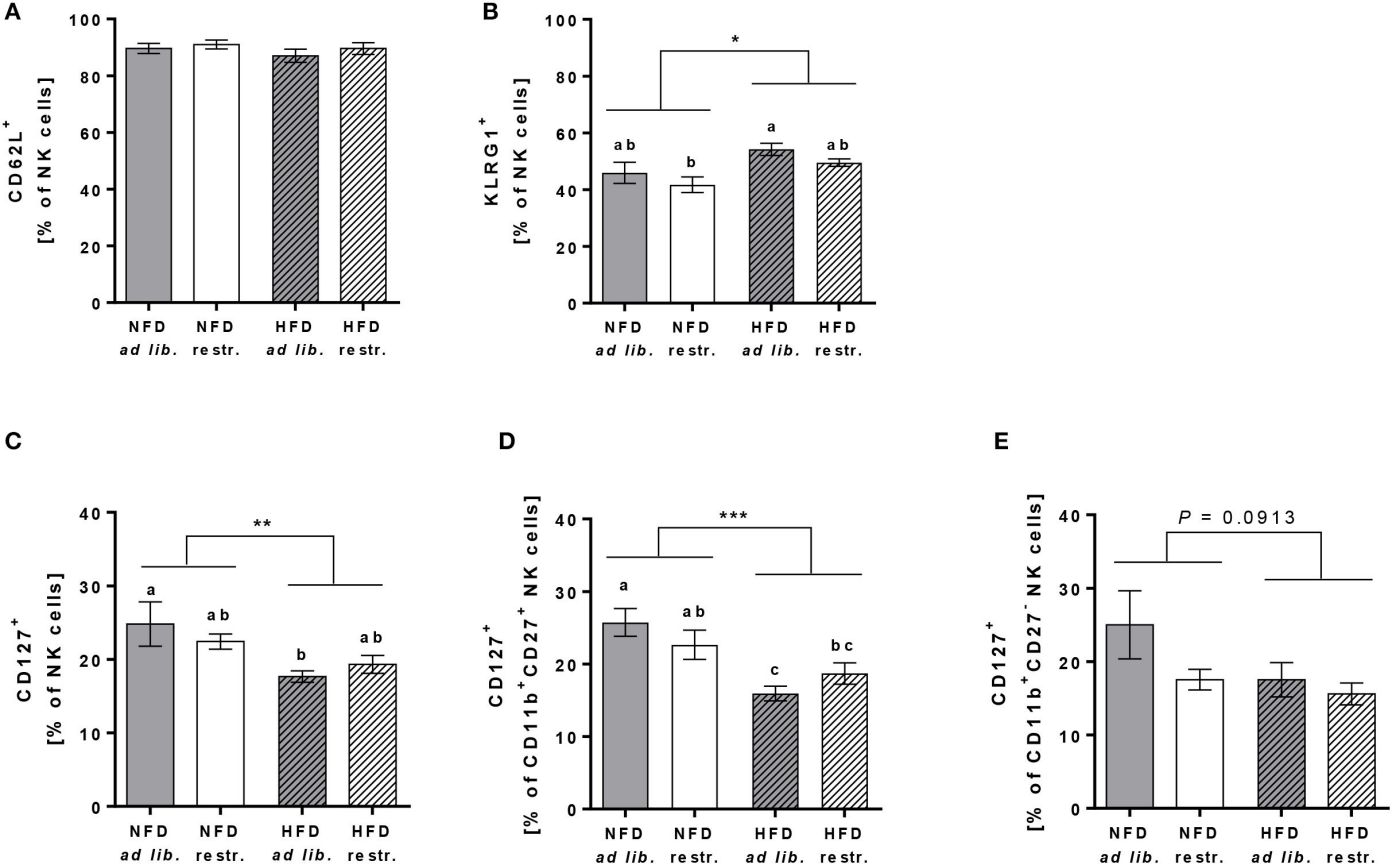
**(A,B)** Flow cytometric analyses of frequencies of total NK cells of C57BL/6 mice expressing the adhesion molecule CD62L **(A)** and the maturation marker killer cell lectin-like receptor G1 (KLRG1, B). **(C–E)** Frequencies of total NK cells, CD11b^+^CD27^+^NK cells and CD11b^+^CD27^−^ NK cells of C57BL/6 mice expressing the maturation marker CD127. **(B–D)** Mice were fed either a high-fat diet (HFD) or normal-fat diet (NFD) under *ad libitum* (*ad lib*.) or restrictive (restr.) feeding regimes. Means with different letters are significantly different according to *post-hoc* Tukey's multiple comparison test results (*P* ≤ 0.05). *P ≤ 0.05, **P ≤ 0.01, ***P ≤ 0.001, two-way ANOVA, NFD fed groups compared to HFD fed groups. Data are presented as mean ± SEM.

In addition, investigations on the expression of the adhesion molecule CD62L on total NK cells demonstrated no differences between the four experimental groups (Figure 6A). In contrast, the frequency of the maturation marker KLRG1^+^ NK cells was significantly increased in mice fed the HFD compared to mice fed the NFD. In addition, mice fed the HFD *ad libitum* had a significantly higher KLRG1 expression on total NK cells compared to mice fed the NFD in a restrictive manner (Figure 6B). Interestingly, the expression of the maturation marker CD127 on total NK cells and CD11b^+^CD27^+^ NK cells was significantly decreased in mice fed the HFD compared to mice fed the NFD independent of the feeding regime (Figures 6C,D). In both, total NK cells and the CD11b^+^CD27^+^ NK cell subset, the decrease of CD127 expression was significant in mice fed the HFD *ad libitum*, but not in mice fed the HFD in a restrictive manner (Figures 6C,D). No significant differences in CD127 expression were detected in the CD11b^+^CD27^−^ NK cell subset (Figure 6E).

### Cytolytic Activity of Splenic NK Cells Against the Murine Colon Cancer Cell Line CT26.WT

For analyses of cytotoxicity of primary murine splenic NK cells against CT26.WT cells, preliminary tests were performed to explore the optimal E:T ratio. Preliminary experiments revealed that no cytolytic effect of NK cells against CT26.WT cells were observed using E:T ratios of 1:1, 4:1, 6:1, 12:1, 16:1, 25:1, 32:1, and 40:1 (data not shown), but a higher E:T ratio of 70:1 was sufficient to detect appropriate cytotoxic effects of NK cells. As it was not possible to isolate the required NK cell number from each mice spleen, only a small number of mice (*n* = 2–4 per group) could be included to use their isolated NK cells with the pre-defined E:T ratio of 70:1.

**Figure 7:**
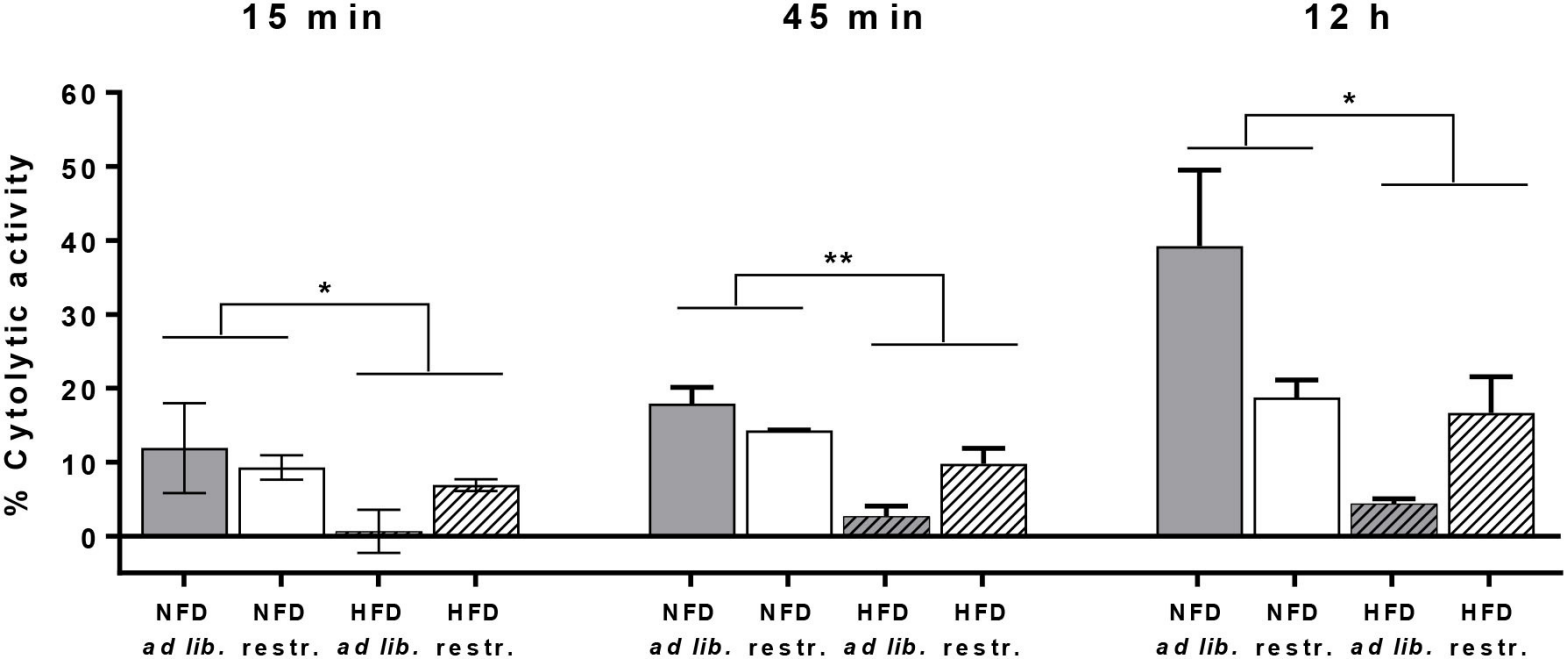
Cytolytic activity of primary splenic NK cells of C57BL/6 mice against the colon cancer target cell line CT26.WT at 15 min, 45 min, and 12 h after NK cell addition determined by the real-time xCELLigence system. Mice were fed a normal-fat diet (NFD) or a high-fat diet (HFD) with both *ad libitum* (ad lib.) and restrictive (restr.) feeding regimes. *P ≤ 0.05 and **P ≤ 0.01 indicate results of two-way ANOVA for the main factor diet. Data are presented as mean ± SEM.; *n* = 2–4 (per group).

Due to the small sample size per group at an E:T of 70:1 it was not applicable to perform statistic analysis on differences between the four mice groups. Therefore, Figure 7 shows the obtained cytolytic activity of splenic NK cells against CT26.WT cells 15 min, 45 min and 12 h after co-incubation without data on intergroup statistics. Results showed a significantly reduced NK cell cytotoxicity in HFD-fed mice compared to NFD-fed mice at the three different time points (Figure 7).

### Gene Expression Analyses of NK Cell Receptors and NK-Cell Related Parameters in Adipose Tissue

To investigate whether HFD or restrictive feeding may also influence functional NK cell markers in adipose tissue, the relative mRNA expression of activating and inhibitory receptors, NK cell-related transcription factors, NKG2D ligands and cytokines were analyzed by real-time RT PCR. Results are delineated in Table 1.

**Table 1:** Real-time polymerase chain reaction (PCR) analyses of effects of high-fat diet (HFD) and normal-fat diet (NFD) on relative mRNA expression of NK cells, activating and inhibitory NK cell receptors, NK cell-related transcription factors, NKG2D-receptor ligands and cytokines in visceral adipose tissue under *ad libitum* and restrictive feeding regimes in C57BL/6 mice.

**Parameter**	**Name and**	**NFD**		**HFD**		**Two-way ANOVA**		
	**alternative names**	**(mean** **±** **SEM)**		**(mean** **±** **SEM)**		**(*****P*****-value)**		
		***ad libitum***	**Restrictive**	***ad libitum***	**Restrictive**	**Diet**	**Feeding regime**	**Diet x feeding regime interaction**
Activating NK cell receptors	Fcgr3 (CD16)	1 ± 0.222^b^	0.626 ± 0.097^b^	5.049 ± 0.801^a^	1.704 ± 0.263^b^	<0.0001	0.0015	0.0089
	Klra4 (Ly49d)	1 ± 0.134	0.993 ± 0.189	1.079 ± 0.201	0.873 ± 0.110	0.9061	0.5312	0.5595
	Klra8 (Ly49h)	1 ± 0.134	0.731 ± 0.069	0.820 ± 0.124	0.770 ± 0.062	0.1864	0.7611	0.9552
	Klra22 (Ly49s2)	1 ± 0.137	0.693 ± 0.063	1.136 ± 0.182	0.754 ± 0.063	0.4760	0.0172	0.7837
	Klrb1c (NK1.1, CD161)	1 ± 0.132^b^	1.297 ± 0.185^ab^	2.007 ± 0.270^a^	1.204 ± 0.140^b^	0.0383	0.2388	0.0140
	Klrk1 (NKG2D)	1 ± 0.138	0.785 ± 0.113	1.161 ± 0.172	0.772 ± 0.097	0.5886	0.0340	0.5274
	NCR1 (NKp46, CD335)	1 ± 0.134	0.976 ± 0.135	1.162 ± 0.152	0.897 ± 0.100	0.7590	0.2899	0.3777
Activation-associated NK cell receptor	CD69	1 ± 0.248	0.637 ± 0.029	0.867 ± 0.126	0.578 ± 0.062	0.4756	0.0207	0.7826
Co-activating NK cell receptor	CD244 (2B4)	1 ± 0.238^ab^	0.780 ± 0.079^b^	1.758 ± 0.353^a^	1.179 ± 0.139^ab^	0.0264	0.1176	0.4760
Inhibitory NK cell receptors	Klra1 (Ly49a)	1 ± 0.132	0.865 ± 0.116	0.803 ± 0.102	0.627 ± 0.051	0.0371	0.1297	0.8365
	Klra2 (Ly49b)	1 ± 0.118^ab^	0.825 ± 0.088^b^	1.569 ± 0.173^a^	1.407 ± 0.197^ab^	0.0024	0.341	0.9726
	Klra3 (Ly49c)	1 ± 0.170	0.828 ± 0.145	1.154 ± 0.213	0.747 ± 0.053	0.8226	*0.0828*	0.4715
	Klra5 (Ly49e)	1 ± 0.115	0.719 ± 0.075	1.049 ± 0.196	0.702 ± 0.052	0.9052	0.0269	0.8101
	Klra6 (Ly49f)	1 ± 0.164	0.688 ± 0.087	0.877 ± 0.178	0.537 ± 0.058	0.3253	0.0235	0.9199
	Klra7 (Ly49g)	1 ± 0.111	0.792 ± 0.146	0.898 ± 0.102	0.661 ± 0.064	0.2777	0.0430	0.8886
	Klra9 (Ly49i)	1 ± 0.159	0.761 ± 0.098	1.014 ± 0.172	0.734 ± 0.065	0.9788	*0.0665*	0.8957
	Klra10 (Ly49j)	1 ± 0.125^ab^	0.743 ± 0.091^ab^	1.195 ± 0.183^a^	0.710 ± 0.059^b^	0.5466	0.0088	0.3947
	Klrc1 (NKG2A)	1 ± 0.206	0.608 ± 0.058	1.107 ± 0.167	0.802 ± 0.113	0.3261	0.0274	0.7750
	Klrd1 (CD94)	1 ± 0.150^ab^	0.841 ± 0.090^b^	1.542 ± 0.218^a^	1.026 ± 0.084^ab^	0.0288	0.0413	0.2684
Co-inhibitory NK cell receptor	PDCD1 (PD-1, CD279)	1 ± 0.062^ab^	0.836 ± 0.130^b^	1.537 ± 0.232^a^	0.990 ± 0.061^ab^	*0.0518*	0.0456	0.2681
Transcription factors	Eomes	1 ± 0.284^ab^	0.629 ± 0.040^b^	1.268 ± 0.169^a^	0.704 ± 0.057^b^	0.2930	0.0065	0.5507
	Tbx21 (T-bet)	1 ± 0.097^ab^	0.848 ± 0.059^b^	1.853 ± 0.35^a^	0.873 ± 0.106^b^	*0.0708*	0.0220	*0.0873*
NKG2D receptor ligands	Ulbp1 (MULT-1)	1 ± 0.117^b^	0.739 ± 0.051^b^	5.292 ± 0.67^a^	2.380 ± 0.255^b^	<0.0001	0.0013	0.006
	Rae1 (RAE-1)	1 ± 0.098	0.817 ± 0.114	0.775 ± 0.081	0.795 ± 0.081	0.2302	0.4275	0.3225
Cytokines	TNF-α	1 ± 0.205^b^	0.568 ± 0.067^b^	3.821 ± 0.652^a^	2.232 ± 0.475^ab^	0.0002	*0.0618*	0.2757
	TNFsf10 (TRAIL)	1 ± 0.177	0.770 ± 0.126	0.662 ± 0.057	0.642 ± 0.087	0.0406	0.2610	0.3440

*CD, cluster of differentiation; Eomes, eomesodermin; Fcgr-3, Fc gamma receptor-3; HFD, high-fat diet; Klr, killer cell lectin-like receptor; MULT-1, mouse UL16-binding protein-like transcript 1; NCR, natural cytotoxicity receptor; NFD, normal-fat diet; NKG, natural killer group; PCR, polymerase chain reaction; PD-1, programmed cell death receptor-1; Ppia, peptidylprolyl isomerase A; Rae-1, retinoic acid early inducible-1 gene; SEM, standard error of the mean; T-bet, T-cell associated transcription factor; Tbx21, T-box transcription factor 21; TNF, tumor necrosis factor; TRAIL, tumor necrosis factor related apoptosis inducing ligand; Ulbp-1, UL16-binding protein−1. Different superscript letters (a, b) indicate significant differences between individual experimental groups analyzed by Tukey's multiple comparison test (P ≤ 0.05). For two-way ANOVA analyses, P-values are shown for the main factors diet, feeding regime and the interaction of both main factors. Significant differences are printed in bold type. Italic P-values indicate a tendency to significance (0.5 ≤ P ≤ 0.1)*.

Investigations on activating NK cell receptors demonstrated that the relative mRNA expression of Klrb1 (CD161) and Fcgr3 (Fc gamma receptor-3; CD16) in adipose tissue was significantly increased in mice fed the HFD *ad libitum* compared to mice fed the NFD *ad libitum*, whereas no effects were observed between mice fed the NFD and HFD in a restrictive manner. In addition, mice fed the HFD restrictively displayed significant lower relative mRNA concentrations of Klrb1 (CD161) and Fcgr3 (CD16) compared to mice fed the HFD *ad libitum*. Moreover, results demonstrated that the mRNA of the activating NK cell receptors Klrk1 (NKG2D) and Klra22 (Ly49s2) as well as for the activation-associated NK cell receptor CD69 in adipose tissue was significantly decreased by restrictive feeding regimes independent of the diet. Results showed a significantly reduced mRNA expression levels of the co-activating receptor CD244 (2B4) in mice fed the HFD compared to mice fed the NFD independent of the feeding regime. Analyses of CD244 (2B4) in single groups revealed significant increased mRNA expression levels in mice fed the NFD restrictively compared to mice fed the HFD *ad libitum*.

Analyses of inhibitory NK cell receptors demonstrated an increased mRNA expression of Klrd1 (CD94) and Klra2 (Ly49b) in adipose tissue of mice fed the HFD compared to mice fed the NFD independent of the feeding regime. For both markers, analyses in single groups revealed significant increased mRNA expression levels mice fed the HFD *ad libitum* compared to mice fed the NFD restrictively. Moreover, results showed a significantly decreased Klrd1 (CD94) expression in restrictive-fed mice compared to *ad libitum*-fed mice independent of the diet. This decreasing effect of the restrictive feeding was also observed for the mRNA expression of the inhibitory NK cell receptors Klra5 (Ly49e), Klra6 (Ly49f), Klra7 (Ly49g), Klra10 (Ly49j), Klrc1 (NKG2A), and the co-inhibitory receptor PDCD1 (PD-1, programmed cell death receptor-1). In addition, mice fed the HFD restrictively displayed significantly reduced Klra10 (Ly49j) mRNA concentrations compared to mice fed the HFD *ad libitum*. Expression levels of PDCD1 (PD-1) mRNA was significantly higher in mice fed the HFD *ad libitum* compared to mice fed the NFD in a restrictive manner. Results showed significantly reduced mRNA expression of the inhibitory NK cell receptor Klra1 (Ly49a) in mice fed the HFD compared to mice fed the NFD independent of the feeding regime.

Investigations on transcription factors in adipose tissue revealed a significant reduction of Eomes (eomesodermin) and Tbx21 (T-box transcription factor 21; T-bet, T-cell associated transcription factor) expression by restrictive feeding independent of the diets. Analyses in single groups revealed significantly lower relative mRNA expression levels of Eomes and Tbx21 (T-bet) in mice fed the HFD restrictively compared to mice fed the HFD *ad libitum*. Moreover, the relative mRNA expression of Eomes and Tbx21 (T-bet) is significantly increased in the HFD *ad libitum* group compared to the NFD restrictive group.

Analyses of the NKG2D receptor ligand Ulbp1 (UL16-binding protein−1; MULT-1, mouse UL16-binding protein-like transcript 1) expression demonstrated significantly higher mRNA concentrations in HFD-fed mice compared to NFD-fed mice independent of the feeding regime. The relative MULT-1 mRNA expression was significantly increased in the HFD *ad libitum* group compared to all other experimental groups.

Analyses of the cytokine TNFsf10 (TRAIL) demonstrated significantly decreased relative mRNA expression levels in mice fed the HFD compared to mice fed the NFD independent of the feeding regime. In contrast, HFD-fed mice revealed significantly increased mRNA concentrations of the cytokine TNF-α compared to NFD-fed mice independent of the feeding regime. Single-group analyses demonstrated that this increase was significant in *ad libitum-*fed mice, but not in restrictive-fed mice receiving the HFD.

No significant differences between the experimental groups were observed for the activating NK cell receptors Klra4 (Ly49d), Klra8 (Ly49h) and NCR1 (NKp46, CD335), for the inhibitory NK cell receptors Klra3 (Ly49c) and Klra9 (Ly49i) as well as for the NKG2D receptor ligand Rae1 (RAE-1, retinoic acid early inducible-1 gene).

## Discussion

Underlying mechanisms for the increased susceptibility to infections and the higher risk for several cancer types in obesity have been widely investigated. Results of previous animal studies demonstrated that overall obesity is associated with an impaired NK cell physiology (18, 19, 21, 39). However, results of those studies were partially conflicting and may be influenced by using different animal species and strains, different feeding periods or composition of high-fat diets as well as the use of different markers and methods to identify NK cells and subsets in blood and different tissues (38). In this study, we investigated the impact of obesity on NK cells in C57BL/6 mice which were exposed to either a normal fat or high-fat diet in an *ad libitum* or restrictive feeding regime for the first time. A secondary aim of the dietary restriction of the normal fat diet was to mimic the natural restricted access to food of wild mice and compare it to an unlimited access to the high-fat diet, thereby reflecting the abundance of food in the industrialized nations and the resulting increase of obesity. In line, recent studies have shown that intermitting fasting, symbolized by the restricted feeding regimes in our study, can positively impact on multiple cellular signaling pathways and on cancer control (48).

C57BL/6 mice are widely used for studies on diet-induced obesity in experimental animals. In line with previous results, body weight and visceral fat mass were significantly increased in C57BL/6 mice fed the HFD *ad libitum* and restrictively compared to the NFD-fed group. Although C57BL/6 mice have previously been shown to be partially obesity-resistant, all HFD-fed C57BL/6 mice reached obesity in the present study (45, 46). In addition, in our study, restrictive feeding as compared to *ad libitum* feeding resulted in a significantly decreased body weight and visceral fat mass in HFD-fed C57BL/6 mice. In contrast, NFD-fed C57BL/6 mice did not show this difference with their feeding regimes.

Several studies already demonstrated increased plasma concentrations of pro-inflammatory cytokines in diet-induced obese C57BL/6 mice (49, 50). In congruence, data of the present study also showed increased IL-6 plasma levels in C57BL/6 mice fed the HFD *ad libitum* compared to the NFD-fed *ad libitum* group. Interestingly, the restrictive feeding of HFD led to significantly reduced IL-6 plasma concentrations compared to the HFD *ad libitum* fed group, reaching the basal levels of both NFD-fed animal groups. In accordance with other studies, this indicates that even a moderate restriction of the amount and availability of a HFD can diminish an obesity-induced inflammatory state (51, 52).

Analysis of total cellular blood leukocytes counts revealed significantly enhanced absolute leukocyte number per μl blood in HFD-fed C57BL/6 mice compared to their NFD-fed littermates. Similar and even more pronounced increases in leukocyte and lymphocyte cell counts in the bone marrow and in the peripheral blood of HFD-fed C57BL/6 mice compared to control mice were described by Trottier et al. (53). The determined cellular increase in HFD-fed mice in the present study are mainly caused by the significant increase in counts of B lymphocytes. An increased frequency of B lymphocytes, especially of pro-inflammatory B cells, as well as impaired B cell functions in obesity have also been shown in previous studies (14, 54). Consistent with previous reports, the present results also demonstrated significantly higher total cell counts and frequencies of monocytes in HFD-fed mice compared to NFD-fed mice (53, 55). In obesity, circulating monocytes highly infiltrate into adipose tissue, where they differentiate into macrophages (56, 57). The obesity-related adipose tissue microenvironment leads to a polarization of the differentiation to pro-inflammatory M1 macrophages instead of anti-inflammatory M2 macrophages, causing and enhancing adipose tissue inflammation (58, 59). Granulocytes are comprised of different cell types including neutrophils as the most abundant with more than 90%. As previous studies already determined increased cell counts of neutrophils in HFD fed mice, it can be assumed that the elevated numbers of granulocytes may be caused by an increased number of neutrophils in the present study (53, 60). Neutrophils are considered to be the first type of cells rapidly recruited to sites of inflammation and they are capable to recruit monocytes and facilitate their infiltration into tissues (11). A previous study reported a primary increased recruitment of neutrophils in AT of HFD fed mice, which preceded the infiltration by monocytes/macrophages (61). In sum, the obtained results on blood cell counts in our study, indicate an enhanced haematopoiesis in HFD fed mice as declared and confirmed by Trottier et al. (53). Interestingly, results of the present study showed a decreased frequency of CD4^+^ and CD8^+^ T cells in HFD-fed mice compared to NFD-fed mice. Several studies reported enhanced recruitment and infiltration of CD4^+^ and CD8^+^ T cells into adipose tissue of obese mice and humans, which may contribute to decreased proportional levels of T cells subsets in the blood observed in the present study (62, 63). Interestingly, data obtained that the restrictive feeding led to a partial, but not significant, prevention of decreased T cell frequencies in HFD-fed mice in the present study, indicating again that a restricted intake of HFD already protected mice from significantly adverse effects of a high-fat consumption under an *ad libitum* feeding regime.

Consistent with previous results in mice and humans, the results of the present study showed a reduced frequency of circulating blood total NK cells in mice fed the HFD *ad libitum* or restrictive (19, 20, 40, 64). Interestingly, the number of peripheral blood total NK cells was not significantly different comparing mice fed the HFD with mice fed the NFD in the present study, which may be attributable to the increased total cell count of monocytes, B cells and granulocytes in obese mice. Previous animal studies analysing only one parameter, total cell numbers or frequencies of NK cells, provided conflicting results about NK cells in peripheral blood or tissues (38). Therefore, the parallel measurement of both parameters, total NK cell number and NK cell frequency, would contribute to more comparable results in future investigations. Further classification and analysis of the four murine NK cell subsets demonstrated a significant reduction of the mature CD11b^+^CD27^+^ NK cell subset in HFD-fed mice compared to NFD-fed mice, regardless of the feeding regime. These findings suggest that HFD feeding may be involved in regulating peripheral NK cell differentiation and maturation. Moreover, as the CD11b^+^CD27^+^ NK cell subset was reported to show the highest levels of cytotoxicity, the reduced frequency of this NK cell subset may contribute to the decreased NK cell cytotoxicity observed in the present study (25).

In addition to differences in NK cell frequency and NK cell subsets, results of the present study showed altered NK cell receptor profiles in obese mice. In contrast to previous investigations, the frequency of NK cells expressing the maturation marker KLRG1 was significantly higher in mice fed the HFD compared to NFD-fed mice independent of the feeding regime (65). KLRG1 expression on NK cells is associated with decreased cytotoxicity (66, 67). Therefore, the decreased lytic activity of NK cells against colon cancer cells in obese mice observed in the presents study may be partially caused by an increased expression of KLGR1 on NK cells mice fed the HFD. Moreover, our results demonstrated a significant decrease of the expression of the maturation marker CD127 in NK cells of HFD-fed mice compared to NFD-fed mice. Interestingly, these findings were also observed in CD11b^+^CD27^+^ NK cells, but not in CD11b^+^CD27^−^ NK cells, indicating a subset-specific effect of HFD-feeding on CD127 expression. Murine CD127^+^ NK cells are considered to have homology to the human CD56^bright^ NK cell subset, as they display poor cytolytic potential, but high proinflammatory cytokine production (68). Therefore, the reduced CD127 expression on NK cells of HFD-fed mice may also influence cytokine production of NK cells. As the reduction of CD127 predominantly occurred in *ad libitum*-fed mice, but not in restrictive-fed mice, in the present study, it can be assumed that a restrictive dietary intake can prevent the HFD-induced alteration of CD127 expression on NK cells.

Until now, only few data exist analysing NK cell-related functional marker in adipose tissue of obese individuals (38, 65, 69). Therefore, the mRNA expression of activating and inhibitory NK cell receptors, NK cell-related transcription factors, NKG2D ligands and cytokines was investigated to get insights whether HFD and restrictive feeding may also influence NK cell parameters in adipose tissue in the present study. Relative mRNA expression levels of the activating NK cell receptors Fcgr3 (CD16) and Klrb1 (CD161) were significantly increased in HFD-fed mice compared to NFD-fed mice under *ad libitum* feeding regime. Interestingly, restrictive feeding prevented the HFD-induced increase of Fcgr3 (CD16) and Klrb1 (CD161) mRNA expression. The mRNA expression of the inhibitory NK cell receptors Klra1 (Ly49a), Klra2 (Ly49b), and Klrd1 (CD94) as well as the co-inhibitory receptor PDCD1 (PD-1) was significantly increased in mice fed the HFD compared to mice fed the NFD in *ad libitum* feeding regime. These results indicate an inhibitory effect of *ad libitum* feeding of a HFD on NK cell activity in adipose tissue. Interestingly, restrictive feeding partly or even significantly attenuated these increasing effects on expression levels of the activating receptor Fcgr3 (CD16) as well as the inhibitory receptors Klra2 (Ly49b), Klra 10 (Ly49j), Klrd1 (CD94) and the co-inhibitory receptor PDCD1 (PD-1). Furthermore, the mRNA expression of several parameters, like the activating receptors Klra22 (Ly49s2) and Klrk1 (NKG2D), the activation-associated receptor CD69, the inhibitory NK cell receptors Klrc1 (NKG2A) as well as Klra5, 6, 7, and 10 (Ly49e-g and j), was significantly reduced in restrictively-fed mice compared to *ad libitum*-fed mice independent of the diet. These results clearly demonstrate that a moderate restriction of dietary fat and energy content influences gene expression regulating NK cell activity in adipose tissue.

Results of previous studies already demonstrated a decreased NK cell cytotoxicity in obese animals and humans (38). However, these analyses were performed using leukemia or lymphoma cell lines as target cells to investigate NK cell lytic activity. In the present study, the cytotoxicity of primary murine NK cells from mice fed the HFD and NFD under restrictive and *ad libitum* feeding regimes against the murine colorectal cancer cell line CT26.WT were analysed for the first time. Data demonstrated that splenic NK cells isolated from HFD-fed mice revealed remarkably reduced NK cell mediated target cell lysis of colorectal tumour cells compared to NFD-fed mice. As CT26.WT cells are derived from BALB/c mice and therefore allogenic to C57BL/6 it is even more impressive that a crude high fat feeding leads to a significantly impaired cytotoxicity of NK cells. More robust data with higher numbers of study subjects are needed, however our preliminary results indicate a prevention of the decreased NK cell cytotoxicity by a restrictive feeding of the HFD.

Until now, only few data exist about the influence of obesity on underlying mechanisms of NK cells. Interestingly, recent studies on human NK cells indicate that the reduced cytotoxicity of NK cells in obese healthy blood donors is induced by an impaired death cell receptor-dependent killing pathway of NK cells (70). Future investigations, like *in vivo* killing experiments and detailed analyses of killing pathways are necessary to specify underlying mechanisms leading to the decreased NK cell cytotoxicity in obese mice and humans.

The transcription factors Eomes and T-bet are known to regulate maturation and differentiation as well as cytokine secretion and cytotoxicity of NK cells (71). In the present study, *ad libitum* feeding of a HFD resulted in significantly increased mRNA expression levels of Eomes and Tbx21 (T-bet) compared to the corresponding *ad libitum*-fed NFD control group. Thus, HFD fed in an *ad libitum* feeding regime may influence maturation, differentiation and functionality of NK cells via upregulation of NK cell-specific transcription factors. Once more, the restrictive feeding of the HFD diminished the changes on Eomes and Tbx21 (T-bet) mRNA expression induced by *ad-libitium* feeding of the HFD in the present study. TRAIL is a well-established player in anti-tumour immunity. The ligation of TRAIL on NK cells with TRAIL receptors expressed on target cells is an important mechanism of target cell lysis via induction of apoptosis (72). As relative mRNA expression of TRAIL is significantly decreased in mice fed the HFD compared to mice fed the NFD, it can be assumed that obesity impairs the cytotoxic capacity of adipose tissue NK cells. However, the present findings on functional NK cell-related markers were determined in the total adipose tissue. Of note and important for the before mentioned results, numerous of these analyzed parameters are also expressed on other (immune) cells present in adipose tissue. In addition, results of real-time PCR analyses only display the gene expression of these markers, but not expression of proteins. Therefore, further investigations, e.g., flow cytometry analyses of the stromal vascular fraction, are necessary to verify the potential restriction of findings in adipose tissues to NK cells.

Previous studies demonstrated an increased NK cell number as well as an increased secretion of the pro-inflammatory cytokines IL-6, IFN-γ and TNF-α by NK cells in adipose tissue in diet-induced obese mice and humans (73–76). Moreover, adipose tissue NK cells of obese individuals can induce the polarization from the anti-inflammatory M2 macrophages to the pro-inflammatory M1 macrophages (73). Therefore, NK cells have been discussed to contribute to the obesity-induced adipose tissue inflammation which may contribute to the obesity-induced pathogenesis of insulin resistance and type 2 diabetes mellitus (65, 73). Future studies are necessary to analyze the role of the obesity-associated alterations of NK cell markers observed in this study for adipose tissue inflammation and the development of obesity-induced metabolic disorders.

Bähr et al. (38) interestingly, numerous of the differences on the investigated parameters were most pronounced between mice fed the NFD restrictively and mice fed the HFD *ad libitum*. This indicates that the unlimited access to high-caloric and high-fat food in the industrialized nations may be a potential factor for the observed obesity-related impaired immune cell phenotype and, therefore, the higher cancer risk in obese individuals.

Obesity is a preventable risk factor for numerous diseases including several cancer types.

Previous investigations with adoptive transfer of NKs cells revealed evidence that NK cell physiology is dependent on the metabolic environment as alterations of NK cell phenotype in obese rats could be ameliorated by transfer of NK cells into normal weight rats (77). Moreover, studies confirmed that reduction of body weight and fat mass in obese humans through a combined dietary and exercise program or bariatric surgery can attenuate the obesity-associated alterations of NK cells (78, 79). Results of the present study confirm these findings and clearly demonstrated for the first time, that even a moderate restriction of the amount and availability of a diet rich in energy and fat can partially diminish the obesity-related dysfunctions of NK cells.

In conclusion, results of the present study demonstrate alterations in immune cell populations, a decreased proportional frequency of total NK cells and the mature CD11b^+^CD27^+^ NK cell subset in diet-induced obese mice. Feeding a high-fat diet resulted in increased expression of the maturation markers KLGR1 and CD127 on NK cells of C57BL/6 mice which may lead to decreased cytolytic and immunoregulatory capacities of NK cells. Furthermore, HFD-fed mice revealed various alterations in the mRNA expression of NK cell-related functional marker in adipose tissue. In addition, diet-induced obese mice displayed a reduced cytotoxicity of splenic NK cells against colorectal cancer cells. Interestingly and for the first time, our data clearly demonstrate that a moderate dietary restriction of HFD intake can partially prevent the obesity-associated alterations of NK cells.

## Data Availability Statement

All datasets generated for this study are included in the article/Supplementary Material.

## Ethics Statement

The animal study was reviewed and approved by State administration of Saxony-Anhalt, Animal welfare committee, Dessauer Strasse 70, 06118 Halle (Saale), Germany.

## Author Contributions

IB and JS planned, conducted, supervised the study, and were major contributors in writing the manuscript with support from WN. ME, MA, and WN carried out the animal experiments under supervision of JS and IB. DQ planned and designed the flow cytometric set up, analyses workflow, and conducted the flow cytometry analyses. WN performed NK cell purification and the cytotoxicity assays under supervision of IB and JS. DQ and HK revised the manuscript critically. All authors contributed to the article and approved the submitted version.

## Conflict of Interest

The authors declare that the research was conducted in the absence of any commercial or financial relationships that could be construed as a potential conflict of interest.
