# Supplementary material for: High-Fat Diet and Feeding Regime Impairs Number, Phenotype, and Cytotoxicity of Natural Killer Cells in C57BL/6 Mice
Source: Front Nutr. 2020 Nov 27;7:585693. doi: 10.3389/fnut.2020.585693 (PMC7728990; doi:10.3389/fnut.2020.585693)
Supplement: Supplementary file 1 [file Table_1.pdf]

Supplementary Table 1: Fluorochrome-conjugated mononuclear antibodies for surface staining of murine peripheral blood immune cells for flow cytometric analysis.

**Antigen**	**Fluorochrome**	**Isotype**	**Clone**	**Concentration [µg/ml]**	**Company**
CD3e	PerCP	Hamster IgG1, κ	145-2C11	100.00	BD Biosciences
Ly-6G	BV510	Rat IgG2a, κ	1A8	50.00	BD Biosciences
Ly-6C	BV605	Rat IgM, κ	AL-21	200	BD Biosciences
KLRG1	BV421	Hamster IgG2, κ	2F1	50.00	BD Biosciences
CD8a	Alexa Fluor 700	Rat IgG2a, κ	53-6.7	100.00	BD Biosciences
CD4	PE-Cy	Rat IgG2a, κ	RM4-5	100.00	BD Biosciences
CD45	FITC	Rat IgG2b, κ	30F11	150.00	Miltenyi Biotec
CD27	PE	Hamster IgG	LG.3A10	30.00	Miltenyi Biotec
CD127	PE-Vio770	Rat IgG2aκ	A7R 34	30.00	Miltenyi Biotec
CD335 (NKp46)	APC	Rat IgG2aκ	29A1.4.9	150.00	Miltenyi Biotec
CD161 (NK1.1, NRP1C)	PE-Vio770	Mouse IgG2aκ	PK136	150.00	Miltenyi Biotec
CD122 (IL-2Rβ)	PE-Vio770	Rat IgG2bκ	TM-β1	30.00	Miltenyi Biotec
CD69	PE	Hamster IgG1	H1.2F3	30.00	Miltenyi Biotec
CD314 (NKG2D)	PE-Vio770	Rat IgG1κ	CX5	30.00	Miltenyi Biotec
CD62L	PE	Rat IgG2aκ	MEL14-H2.100	30.00	Miltenyi Biotec
Ly-49C/F/I/H	APC-Vio770	Hamster IgG	14B11	30.00	Miltenyi Biotec
CD244.2 (2B4)	PE-Vio770	Recombinant human IgG1	REA388	30.00	Miltenyi Biotec
CD19	APC-Vio770	Rat IgG2aκ	6D5	30.00	Miltenyi Biotec
CD11b	VioBlue	Recombinant human IgG1	M1/70.15.11.5	33.00	Miltenyi Biotec
CD49b	PE	Rat IgM κ	DX5	30.00	Miltenyi Biotec

APC, allophycocyanin; BV, brilliant violet; CD, cluster of differentiation; Cy, cyanine; FITC, fluorescein isothiocyanate; Ig, immunoglobulin; IL-2R, interleukin-2 receptor; Klr, killer cell lectin-like; NKG2, natural killer group two; PE, phycoerythrin; PerCP, peridinin chlorophyll protein;
BD Biosciences, San Jose, CA, USA; Miltenyi Biotec, Bergisch Gladbach, Germany.

Supplementary Table 2: Parameters of primers used for real-time RT-PCR analyses.

**Gene**	**Primer sequence (5`-3`)**		**Product size (bp)**	**NCBI gene bank reference**
	**Forward**	**Reverse**		
2B4 (CD244)	CAGTATATTCAGTAGTCCAGC	CACGGTACAACTTAAGGAAG	80	NM_018729
CD69	AAAAGGACATGACGTTTCTG	CAGCTGTTAAATTCTTTGCC	115	NM_001033122
Eomes	ACAACACACAGATGATAGTG	TATGGTCGATCTTTAGCTGG	195	NM_001164789
Fcgr3 (CD16)	ATTTCTCTATCCCAAAAGCC	CTAGAGAGATGGAGGATGTAG	138	NM_010188
Klra1 (Ly49a)	ATGGACAGAAAAACATGGAG	TTCAAGGCAATTTAGATGG	200	NM_016659
Klra2 (Ly49b)	CAAGGGAAGAACTCAATCTG	GACACAAGCTTCTCTGTTTAC	120	NM_001170851
Klra3 (Ly49c)	CAGAGGTGTTAAATACTGGTT	ATTCTCTGGAATAACATGGC	172	NM_010648
Klra4 (Ly49d)	TTTCGTCATGGACAGAAAAC	GAACTTCAGTTCATCCTCATC	100	NM_010649
Klra5 (Ly49e)	ACAAGAAATCCACGAAACTC	CTTGGTTTCACTGTACCATC	169	NM_008463
Klra6 (Ly49f)	TGACAATGGCCAATCTAAAC	TCTCTATTCACAGCAGTCTATG	200	NM_008464
Klra7 (Ly49g)	AAGCTCATTGTGATAGCTTG	GCTGAAAAATCGTTATTGCC	82	NM_001110323
Klra8 (Ly49h)	AGATACTGACTGTAATACTCC	TGTTCTTTAACTCTGGTTGG	91	NM_010650
Klra9 (Ly49i)	CGTTCCCATTGTGAAGATAG	GACAATCCAATCCAGTAACTC	87	NM_010651
Klra10 (Ly49j)	CCCATTGTGAAGATAGAAGAT	CATGTCAAGTTTAGATGGGC	144	NM_008459
Klra22 (Ly49s2)	GATAGACAATGAGGATGAAC[illegible]	GTTCAAGGCAAGCTTAGATG	136	NM_053152
Klrb1c (NK1.1, CD161)	AACTGAGATTCCTACTGGAC	TTGTGCCATTTATCCACTTC	105	NM_001159904
Klrk1 (NKG2D)	AGTATTGTGCAACAAGGAAG	TTTTGAGACAACCAGGAAGC	153	NM_033078.4
Klrc1 (NKG2A)	CCCACAGAGATATAAACTACA	TGCTCCTCTTCACTATCTATG	184	NM_001136068
Klrd1 (CD94)	AAGTCTTGGAAAAGAAGCAG	GCATTCCAATCCAGAAAAAG	121	NM_010654
NCR1(CD335, NKp46)	TAGTAACTGGTCTGTATGACA[illegible]	CTTGAGCAGAAAGAATTTGC	125	NM_010746
PDCD1 (PD-1)	ACTAGGGCAATAAAGGGAAC	GAATGAGGAGATTCTAACACC	176	NM_008798
Ppia	CACCGTGTTCTTCGACATCA	TGTCTGCAAACAGCTCGAAG	71	NM_008907.1
Rae-1(RAE-1)	CAATGAAGGATATCGAAGTG	GAAGTTGCCTGGTAAAGTTG	86	NM_175112
Tbx21 (T-bet)	ACGTCTTTACTTTCCAAGAG	GTACATGGACTCAAAGTTCTC	128	NM_019507
TNF-α	CTATGTCTCAGCCTCTTCTC	CATTTGGGAACTTCTCATCC	125	NM_013693
TNFsf10 (TRAIL, CD253)	GAAAAGCAGCTAAGTACTCC	ACGTGGTTGAGGAAATGAATG	189	NM_009425.2
Ulbp-1 (MULT-1)	ACCTGTGTTTATGCAGATTG	CCCATCAATATCGTCTGAAG	154	NM_029975

bp, base pair; CD, cluster of differentiation; Eomes, eomesodermin; Fcgr-3, Fc gamma receptor-3; Klr, killer cell lectin-like receptor; MULT-1, mouse UL16-binding protein-like transcript 1; NCBI, National Center for Biotechnology Information; NCR, natural cytotoxicity receptor; NKG, natural killer group; PD-1, programmed cell death receptor-1; Ppia, peptidylprolyl isomerase A; Rae-1, retinoic acid early inducible-1 gene; T-bet, T-cell associated transcription factor; Tbx21, T-box transcription factor 21; TNF, tumor necrosis factor; TRAIL, tumor necrosis factor related apoptosis inducing ligand; Ulbp-1, UL16-binding protein -1.

Supplementary Table 3: Nutritional data of calculated daily dietary intakes in C57BL/6 mice fed a normal-fat diet (NFD) or a high-fat diet (HFD) with *ad libitum* or restrictive feeding regimes.

	NFD (Mean ± SEM)		HFD (Mean ± SEM)		Two-way ANOVA (P-value)		
Nutritional component	*Ad libitum*	restrictive	*Ad libitum*	restrictive	Diet	Feeding regime	Diet x Feeding regime interaction
Food amount [g/day]	3.060 ± 0.043[a]	2.742± 0.011[b]	2.553 ± 0.035[c]	2.313 ± 0.004[d]	**< 0.0001**	**< 0.0001**	0.1719
Energy [kcal/day]	11.78 ± 0.164[b]	10.56 ± 0.043[c]	13.38 ± 0.180[a]	12.12 ± 0.022[b]	**< 0.0001**	**< 0.0001**	0.8955
Fat [g/day]	0.132 ± 0.002[c]	0.118 ± 0.001[c]	0.891 ± 0.012[a]	0.807 ± 0.002[b]	**< 0.0001**	**< 0.0001**	**< 0.0001**
Protein [g/day]	0.588 ± 0.008[b]	0.527 ± 0.002[c]	0.669 ± 0.009[a]	0.606 ± 0.001[b]	**< 0.0001**	**< 0.0001**	0.8858
Carbohydrate [g/day]	2.060 ± 0.029[a]	1.846 ± 0.008[b]	0.672 ± 0.009[c]	0.608 ± 0.001[d]	**< 0.0001**	**< 0.0001**	**< 0.0001**

HFD, high-fat diet; NFD, normal-fat diet; SEM, standard error of the mean. Different superscript letters (a, b, c, d) indicate significant differences between individual experimental groups analyzed by Tukey´s multiple comparison test ($P \leq 0.05$). For two-way ANOVA analyses, P-values are shown for the main factors diet, feeding regime and the interaction of both main factors. Significant differences are printed in bold type.

Supplementary Table 4: Effects of high-fat diet (HFD) and normal-fat diet (NFD) on body weight, visceral fat mass and plasma cytokine concentrations under *ad libitum* and restrictive feeding regimes in C57BL/6 mice.

	NFD (Mean ± SEM)		HFD (Mean ± SEM)		Two-way ANOVA (P-value)		
	Ad libitum	restrictive	*Ad libitum*	restrictive	Diet	Feeding regime	Diet x Feeding regime interaction
Terminal body weight [g]	28.32 ± 0.735[c]	25.29 ± 0.547[c]	46.28 ± 1.103[a]	37.30 ± 1.072[b]	**< 0.0001**	**< 0.0001**	**0.0004**
Visceral fat mass [g]	0.696 ± 0.081[b]	0.553 ± 0.035[c]	3.128 ± 0.168[a]	2.478 ± 0.176[b]	**< 0.0001**	**0.0124**	*0.0980*
Plasma IL-2 concentration [pg/ml]	0.762 ± 0.398	1.491 ± 0.279	1.236 ± 0.481	0.481 ± 0.272	0.4884	0.9740	*0.0628*
Plasma IL-6 concentration [pg/ml]	1.515 ± 0.435[b]	3.301 ± 0.784[b]	9.006 ± 2.021[a]	2.626 ± 0.415[b]	**0.0198**	0.1059	**0.0063**
Plasma IFN-γ concentration [pg/ml]	8.307 ± 1.076	8.499 ± 1.120	11.107 ± 1.221	8.633 ± 0.986	0.2086	0.3251	0.2521
Plasma TNF-α concentration [pg/ml]	1.397 ± 0.240	2.410 ± 0.532	2.159 ± 0.205	1.443 ± 0.086	0.7342	0.6241	**0.0077**

HFD, high-fat diet; IL, interleukin; IFN, interferon; NFD, normal-fat diet; SEM, standard error of the mean; TNF, tumor-necrosis-factor. Different superscript letters (a, b) indicate significant differences between individual experimental groups analyzed by Tukey´s multiple comparison test ($P \leq 0.05$). For two-way ANOVA analyses, P-values are shown for the main factors diet, feeding regime and the interaction of both main factors. Significant differences are printed in bold type. Italic P-values indicate a tendency to significance ($0.05 \leq P \leq 0.1$).
