# Supplementary material for: High-Fat Diet and Feeding Regime Impairs Number, Phenotype, and Cytotoxicity of Natural Killer Cells in C57BL/6 Mice
Source: Front Nutr. 2020 Nov 27;7:585693. doi: 10.3389/fnut.2020.585693 (PMC7728990; doi:10.3389/fnut.2020.585693)
Supplement: Supplementary file 2 [file Data_Sheet_1.PDF]

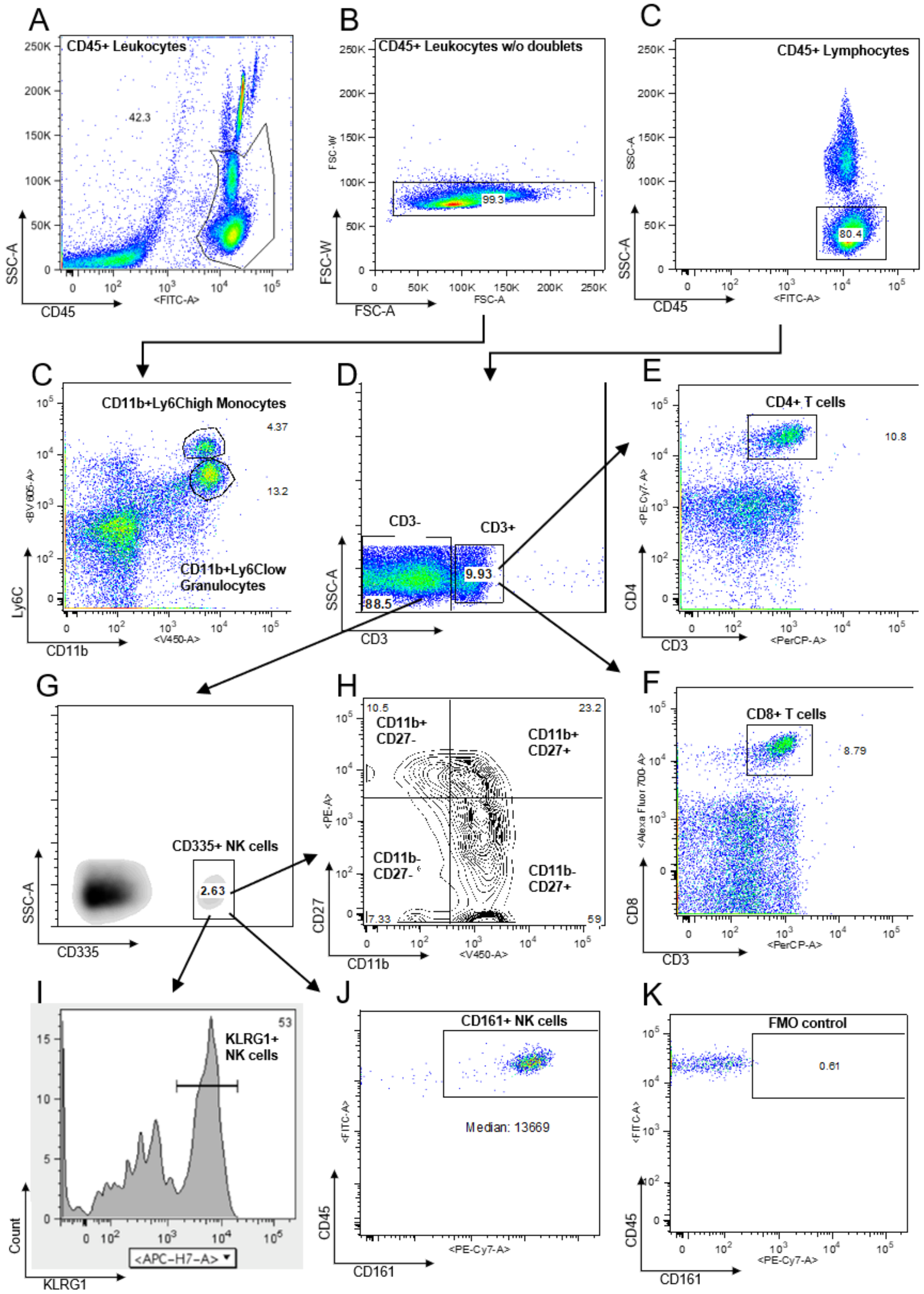

Supplementary Figure 1: Representative plots of the hierarchical gating strategy to identify immune cell populations, NK cell subsets and NK cell surface marker expression from C57BL/6 mice. Leucocytes were identified by their CD45 expression and granularity using SSC (A), followed by doublet exclusion (B), and gating of $CD45^{+}$ SSC^{low} lymphocytes (C). Leucocyte gating combined with single cell gating leads to the identification of $CD11b^{+}Ly6C^{high}$ monocytes and $CD11b^{+}Ly6C^{low}$ granulocytes (D). Based on the lymphocyte gate, $CD3^{-}$ and $CD3^{+}$ lymphocytes (E) were classified. $CD3^{+}CD4^{+}$ helper T cells (E) and $CD3^{+}CD8^{+}$ cytotoxic T cells (F) were identified from the $CD3^{+}$ fraction. $CD335^{+}$ NK cells were identified from the $CD3^{-}$ fraction (G). On the basis of the expression of CD11b and CD27, the total NK cell population was further differentiated into four NK cell subsets (H). The expression of different NK cell marker were assessed on the $CD335^{+}$gated NK cell fraction. Representative images of KLRG as histogram (I) and of CD161 using dot plot are given (J). FMO controls were used to determine positive cells, exemplified for CD161 staining (K).
