# Supplementary material for: High-Fat Diet and Feeding Regime Impairs Number, Phenotype, and Cytotoxicity of Natural Killer Cells in C57BL/6 Mice
Source: Front Nutr. 2020 Nov 27;7:585693. doi: 10.3389/fnut.2020.585693 (PMC7728990; doi:10.3389/fnut.2020.585693)
Supplement: Supplementary file 3 [file Data_Sheet_2.PDF]

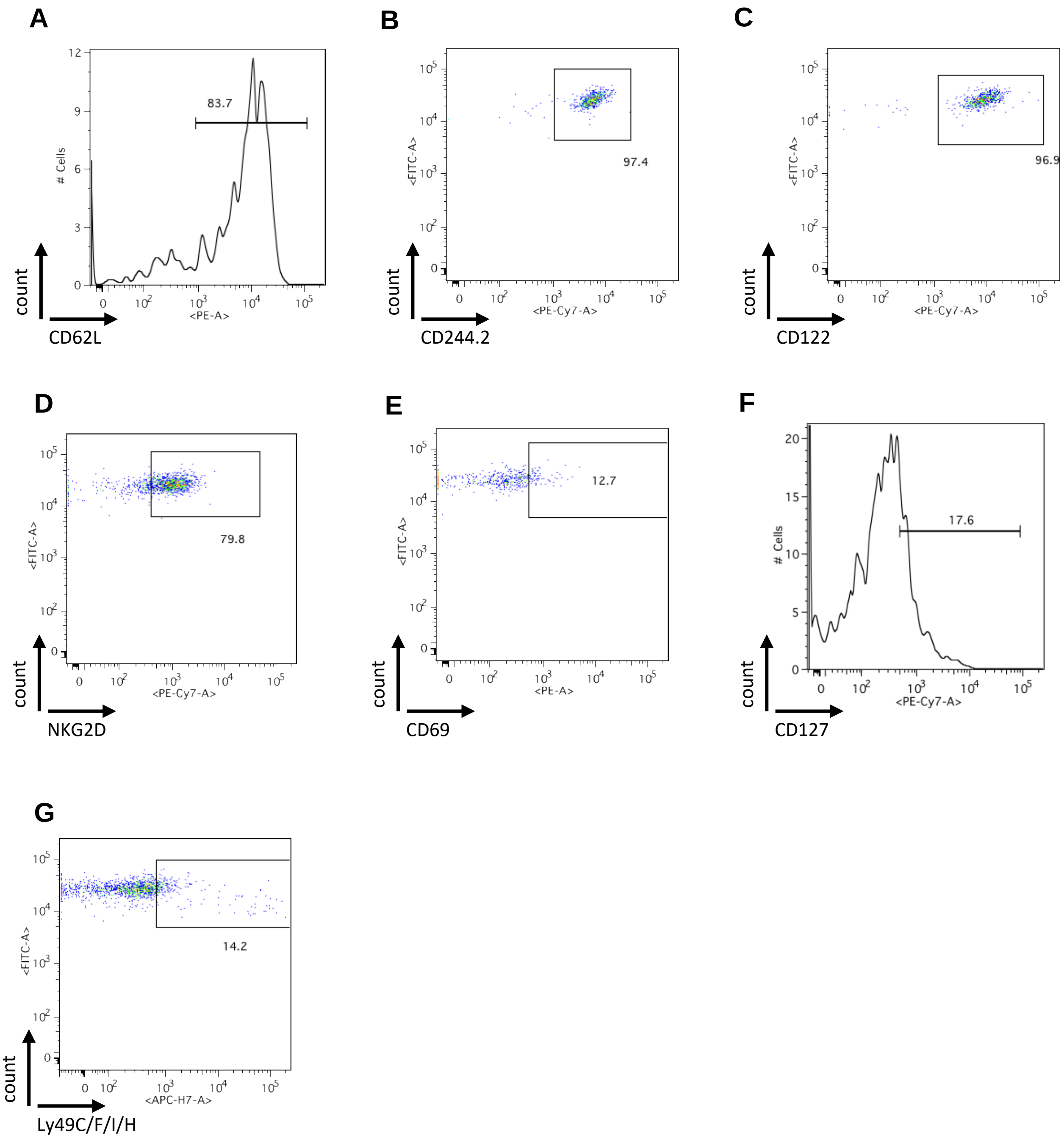

Supplementary Figure 2: Representative flow cytometry images of surface marker expression gated on CD335$^+$ blood NK cells of C57/BL6 mice. Cells were stained in different panels always using the same backbone marker panel with same fluorochromes to identify NK cells. Positive events were determined by the use of FMO controls and visual inspections. CD62L (A), CD244.2 (B), CD122 (C), NKG2D (D), CD69 (E), CD127 (F), Ly49C/F/I/H (G).
