# Supplementary material for: High-Fat Diet and Feeding Regime Impairs Number, Phenotype, and Cytotoxicity of Natural Killer Cells in C57BL/6 Mice
Source: Front Nutr. 2020 Nov 27;7:585693. doi: 10.3389/fnut.2020.585693 (PMC7728990; doi:10.3389/fnut.2020.585693)
Supplement: Supplementary file 4 [file Data_Sheet_3.PDF]

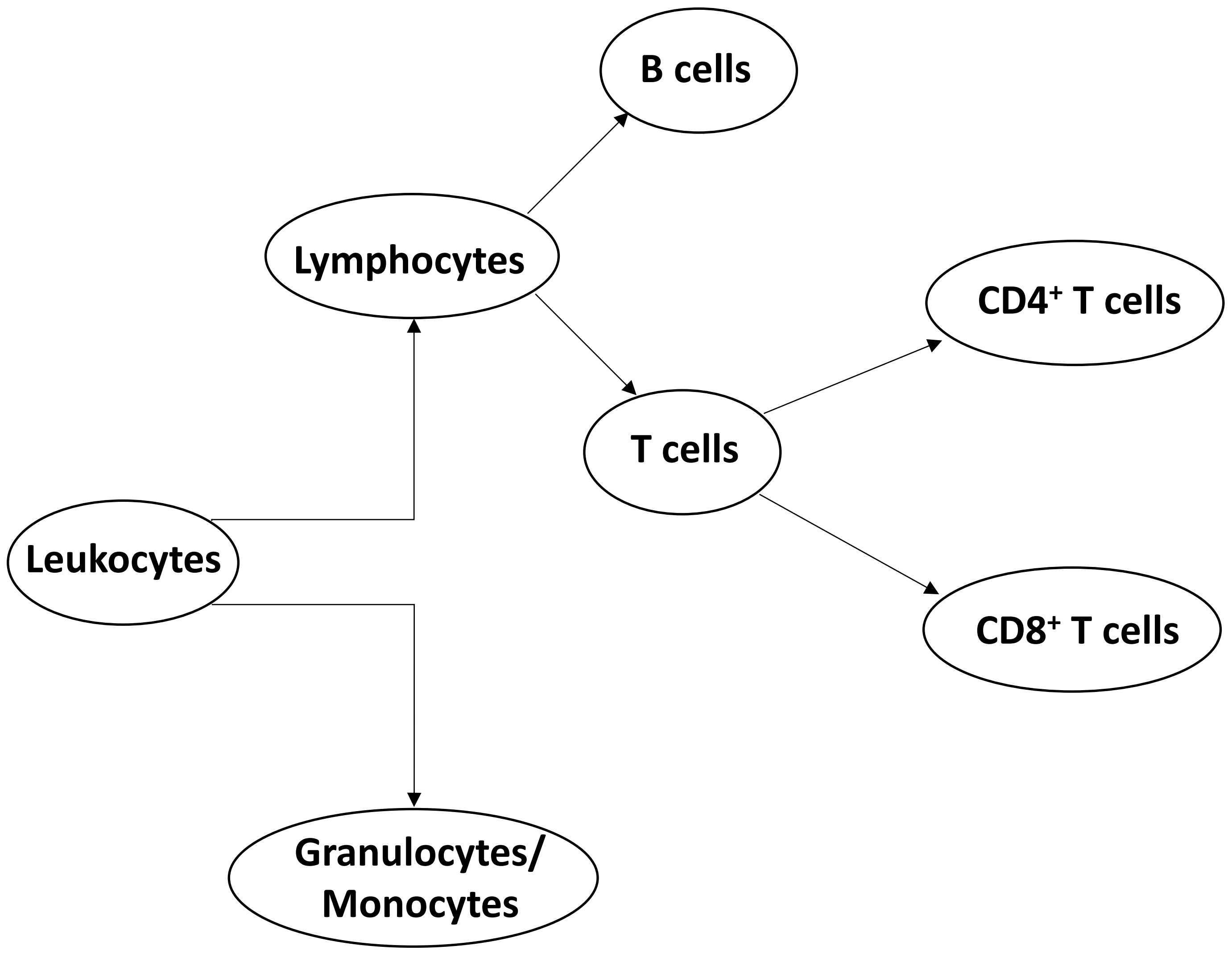

Supplementary Figure 3: Hierarchical pathways of flow cytometric analyses of immune cell populations in C57BL/6 mice. Granulocytes, monocytes and total lymphocytes were determined based on the $CD45^{+}$ leucocytes. B cells and total T cells were defined based on the lymphocyte gate. $CD4^{+}$ helper T cells and $CD8^{+}$ cytotoxic T cells were identified based on the total T cell gate.
